# Development of a novel, entirely herbal-based mouthwash effective against common oral bacteria and SARS-CoV-2

**DOI:** 10.1186/s12906-023-03956-3

**Published:** 2023-05-01

**Authors:** Bálint Bencze, Viktória Temesfői, Sourav Das, Henrietta Papp, Péter Kaltenecker, Anett Kuczmog, Ferenc Jakab, Béla Kocsis, Tamás Kőszegi

**Affiliations:** 1 Department of Laboratory Medicine, Clinical Centre, Medical School, University of Pécs, Ifjúság Út 13, Pécs, 7624 Hungary; 2 Lab-On-a-Chip Research Group, János Szentágothai Research Centre, University of Pécs, Ifjúság Útja 20, Pécs, 7624 Hungary; 3 Hungarian National Laboratory On Reproduction, University of Pécs, Pécs, 7624 Hungary; 4 National Laboratory of Virology, University of Pécs, Ifjúság Útja 20, Pécs, 7624 Hungary; 5 Institute of Biology, Faculty of Sciences, University of Pécs, Ifjúság Útja 6, Pécs, 7624 Hungary; 6 Department of Medical Microbiology and Immunology, Clinical Centre, Medical School, University of Pécs, Szigeti Út 12, Pécs, 7624 Hungary

**Keywords:** Herbal mouthwash, Oral rinse, Oral care, SARS-CoV-2, Essential oil

## Abstract

**Background:**

Parallel to the growth of the oral healthcare market, there is a constantly increasing demand for natural products as well. Many customers prefer products that contain fewer toxic agents, therefore providing an environmentally friendly solution with the benefit of smaller risk to the user. Medieval and early modern medicinal knowledge might be useful when looking for natural, herbal-based components to develop modern products. Along with these considerations we created, tested, and compared an entirely natural mouthwash, named Herba Dei.

**Methods:**

The manufacturing procedure was standardized, and the created tincture was evaluated by GC/MS analysis for active compounds, experimentally tested in cell-based cytotoxicity, salivary protein integrity, cell-free antioxidant activity, anti-bacterial and anti-viral assays, and compared with three market-leading mouthwashes.

**Results:**

Our tincture did not show significant damage in the cytotoxicity assays to keratinocyte and Vero E6 cells and did not disrupt the low molecular weight salivary proteins. Its radical scavenging capacity surpassed that of two tested, partly natural, and synthetic mouthwashes, while its antibacterial activity was comparable to the tested products, or higher in the bacterial aerobic respiratory assay. The active compounds responsible for the effects include naturally occurring phenylpropanoids, terpenes, and terpenoids. Our mouthwash proved to be effective in vitro in lowering the copy number of SARS-CoV-2 in circumstances mimicking the salivary environment.

**Conclusions:**

The developed product might be a useful tool to impede the transmission and spread of SARS-CoV-2 in interpersonal contact and aerosol-generating conditions. Our mouthwash can help reduce the oral bacterial flora and has an antioxidant activity that facilitates wound healing and prevents adverse effects of smoke in the oral cavity.

**Supplementary Information:**

The online version contains supplementary material available at 10.1186/s12906-023-03956-3.

## Background

Nowadays, besides the classical instruments of oral care, like toothbrushes and toothpaste, the use of supplementary tools and materials shows an increasing tendency. Dental floss, interdental toothbrush, and mouthwash can be used to improve oral hygiene [1].

The two most common dental diseases, caries, and periodontal diseases are bacteria-associated illnesses [2–5]. It is necessary to reduce the pathogenic oral flora, however, toothpaste might not contain sufficient antibacterial agents and in the oral cavity, the majority of bacteria can be found on the tongue and on the pharynx, which cannot be cleaned mechanically efficiently [6]. Also, the matured biofilm on the teeth is a resistant plaque that can be effectively eliminated by rinsing after a mechanical cleaning [5, 7–9]. It is beneficial to use agents which decrease the bacterial populations. These agents can be added to mouthwashes thus, by rinsing, most of the surfaces in the oral cavity can be reached and cleaned. The optimal decrease in bacterial populations can be achieved by synthetic and natural active compounds [10–12]. The intended use of mouthwash solutions is to hinder bacterial growth, quorum sensing, and biofilm formation on the oral surfaces [13, 14]. The mechanisms of action include toxicity, membrane disruption, oxidative and acidic stress, interference with carbohydrate metabolism and protein translation [15–17]. Although, eradicating all the germs is impossible and would be also a blast because it could lead to opportunistic infections in the long term. Using a strong disinfection procedure before surgical or other invasive treatments are necessary but carrying it out frequently might be dangerous [18–20].

A mouthwash must be a versatile product; besides killing germs, it must provide fresh breath, anesthetize wounds, or help heal them with an astringent effect without discoloring the teeth or harming the salivary proteins [21–24]. Moreover, to decrease the toxic load of our body, it is beneficial to have a daily-used rinse without any coloring agents, preservatives, or additives that might be swallowed or taken up through the mucosa or wounds. An optimal mouthwash should help to prevent serious issues in the oral cavity and must be investigated precisely [25].

Accordingly, this study aimed at the production of a completely organic herbal-based mouthwash, considering its safety, anti-SARS-CoV-2, and both antibacterial and antioxidant activities.

## Methods

### Research of the current market and the traditional literature

To get a complete picture of the ingredients and active compounds of mouthwashes that are available over the counter, the Hungarian market was surveyed.

The recent statistics about personal care product usage in Hungary in 2021 was accessed at Statista, Ltd. [26]. We also visited 22 pharmacies to trace down the best-selling products. All the selected mouthwashes which represent the market can be bought in pharmacies and drugstores in Hungary and Central Europe. The hazard of their additives was evaluated by the Environmental Working Group (EWG) hazard score system [27]. EWG is a non-profit organization, their Skin Deep database about the cosmetics components combines product ingredient data with information from toxicity and regulatory databases [28]. It uses a color-coded score system; 0–2 (green), 3–6 (yellow), and 7–10 (red) codes for low, moderate, and high hazard ingredients. An online survey was also performed to learn whether this is a real market gap in Hungary currently, or there is no need for a new natural alternative in oral care (Additional File 1). Since there were no entirely natural alternatives on the market, we aimed to process the monastic, folk, and traditional literature for possible ingredients. Four main works from the early modern Hungarian literature were studied to determine the possible composition of the prepared mouthwash. The processed works were the followings; Péter Melius Juhász: *Herbárium* [29], Ferenc Pápai Páriz: *Pax Corporis* [30], József Csapó: *Új füves és virágos magyar kert* [31], translated as “New grassy and flowered Hungarian garden”, a summary of medical botany, and Mihály Kováts military doctor’s book, *Magyar Patika* [32]*,* translated as “Hungarian Pharmacy”.

In consideration of what requirements a mouthwash should fulfill, a list of plants was set up based on literature data, that could be suitable as ingredients. An optimal rinse is antibacterial, antifungal, has wound healing, breath freshening, antioxidant, and anti-inflammatory activity, contains only natural ingredients, has a nice taste and color, and the pH suites the oral environment without dissolving the enamel and harming the salivary proteins.

### Preparation

The mixtures were made using the following dried herbs that were purchased from a local producer, who has the appropriate permissions and/or licenses to produce and sell spice and herb products: 4 g *Calendula officinalis* (prod.nr.: H-110/19, Herbária Ltd., Budapest, Hungary)*,* 1 g *Hibiscus rosa-sinensis* (prod.num.: H-056/20, Herbária Ltd., Budapest, Hungary), 1 g *Hypericum perforatum* (prod.num.: H-101/20, Herbária Ltd., Budapest, Hungary)*,* 2 g *Juglans regia* (prod.num.: H-116/20, Herbária Ltd., Budapest, Hungary)*,* 1.5 g *Lavandula officinalis* (prod.num.: H-136/20, Herbária Ltd., Budapest, Hungary)*,* 2 g *Mentha spicata* (prod.num.: H-076/20, Herbária Ltd., Budapest, Hungary), 0.3 g *Pimpinella anisum* (prod.num.: H-161/19, Herbária Ltd., Budapest, Hungary)*,* 0.2 g *Quercus robur* (prod.num.: H-145/03, Herbária Ltd., Budapest, Hungary)*,* 2 g *Sambucus nigra* (prod.num.: H-139/13, Herbária Ltd., Budapest, Hungary)*.* The plant material was reidentified by Bálint Bencze, dentist, oral surgeon, and phytotherapy specialist. No voucher specimen has been deposited in a publicly available herbarium. Plant materials used in this study can be purchased from the producer. Sample quality is ensured by a standardized management system operated by the producer which complies with the International Organization for Standardization (ISO) 9001 and with the International Featured Standards (IFS) requirements (Additional File 2). The characteristics and the applied parts of the selected plants were summarized in Table 1.

**Table 1:** Characteristics and applied parts of the selected plants

**Effect**	**Name of the herb**	**Family**	**Applied part**	**Main active components**	**References**
Antibacterial	*Lavandula officinalis*	Lamiaceae	Flos	linalool, linalyl acetate, terpinen-4-ol, acetate lavandulol, oci-mene, cineole	[33–35]
	*Mentha spicata*	Lamiaceae	Folium	carvone, limonene, cis-dihydrocarvone, 1,8-cineole	[33, 36, 37]
	*Cinnamomum zeylanicum*	Lauraceae	Aetheroleum	cinnamaldehyde, eugenol	[38]
	*Eugenia caryophyllata*	Myrtaceae		eugenol, trans-caryophyllene, eugenol acetate	[39]
	*Hypericum perforatum*	Hypericaceae	Herba	hyperforin, naphthodianthrones, flavonoids, prenylated phloroglucinols, tannins, volatile oils	[40]
Antifungal	*Cinnamomum zeylanicum*	Lauraceae	Aetheroleum	cinnamaldehyde, eugenol	[38, 41]
	*Eugenia caryophyllata*	Myrtaceae		eugenol, trans-caryophyllene, eugenol acetate	[39]
Breath freshening	*Pimpinella anisum*	Apiaceae	Fructus	trans-anethole, estragole	[42]
Wound healing	*Juglans regia*	Juglandaceae	Folium	caffeoylquinic acids, coumaroylquinic acids, quercetin	[43]
	*Calendula officinalis*	Asteraceae	Petalum	flavonoids, triterpenoids	[44]
Natural preservative	*Quercus robur*	Fagaceae	Cortex	tannins	[45]
Taste	*Eugenia caryophyllata*	Myrtaceae	Aetheroleum	eugenol, trans-caryophyllene, eugenol acetate	[46]
	*Cinnamomum zeylanicum*	Lauraceae		cinnamaldehyde, eugenol	[47]
	*Lavandula officinalis*	Lamiaceae	Flos	linalool, linalyl acetate, terpinen-4-ol, acetate lavandulol, oci-mene, cineole	[48]
	*Menta spicata*	Lamiaceae	Folium	carvone, limonene, cis-dihydrocarvone, 1,8-cineole	[49]
Color	*Hibiscus rosa-sinensis*	Malvaceae	Flos	tannins, anthraquinones, quinines, phenols, flavonoids, alkaloids, terpenoids, saponins	[50]
Antioxidant	*Hypericum perforatum*	Hypericaceae	Herba	hyperforin, naphthodianthrones, flavonoids, prenylated phloroglucinols, tannins	[51]
Anti-inflammatory	*Hypericum perforatum*	Hypericaceae	Herba	hyperforin, naphthodianthrones, flavonoids, prenylated phloroglucinols, tannins, volatile oils	[52]
	*Sambucus nigra*	Sambucaceae	Flos	phenolic acids, flavonoids	[53]
	*Calendula officinalis*	Asteraceae	Petalum	flavonoids, triterpenoids	[54, 55]

A total of 14 g of the prepared mixture was homogenized and deposited in dark glasses. We decided to use 50% ethanol as a solvent because it can extract most of the active ingredients but not the chlorophylls [56, 57]. 1000 mL of 50% ethanol was added to the mixture. The solution was shaken at room temperature for 4 × 30 min daily with ultrasound for 7 days [58]. After centrifugation, the extract was filtered sequentially through an 8–20 μm (AF15), a 1.5–3 μm (AF71H), and a 0.2–0.4 μm (AFST140) pore size filters (Filtrox, St. Gallen, Switzerland). Finally, 250 mL distilled water was added to the solution that was shaken for 6 h [59]. The ethanol content at the end was 40%. 1250 μL of *Cinnamomum zeylanicum* (prod.num.: P370, Aromax Ltd., Budapest, Hungary) and 1200 μL of *Eugenia caryophyllata* (prod.num.: P426, Aromax Ltd., Budapest, Hungary) essential oils (EOs) were added to 1 L of the previously prepared solution. Both essential oils were produced with the steam distillation method and have a quality license in point of active compound content in accordance with the Ph. Hg. VII (Additional File 3). The completed tincture was named Herba Dei (HD) mouthwash concentrate. The production of the mixture was standardized according to the above protocol. The concentrated tincture showed a pH of 5.5–6. The final developed product was registered at the European Commission cosmetic products notification portal (CPNP) under the reference number CPNP1760385. The tested samples were stored in the dark at 4 °C until the analyses.

The EWG scoring for the selected ingredients in our product is summarized in Table 2.

**Table 2:** Classification of the ingredients of HD by EWG [28]

**Mouthwash**	**Manufacturer**	**Ingredients**	**EWG number**
**Herba Dei**	Concordia Duo Ltd	*Hibiscus rosa-sinensis*	1
		*Hypericum perforatum*	1
		*Juglans regia*	1
		*Lavandula officinalis*	1
		*Mentha spicata*	1
		*Pimpinella anisum*	1
		*Quercus robur*	1
		*Sambucus nigra*	1
		*Calendula officinalis*	1
		*Cinnamomum zeylanicum*	3
		*Eugenia caryophyllata*	4
		Ethanol	1

### Gas chromatography/mass spectrometry (GC/MS)

The main oily compounds of the tincture were determined with GC/MS using an Agilent 6890 N analyzer with the 5973N mass-selective detector and an HP-5MS capillary column (30 m × 250 μm × 0.25 μm) (Agilent Technologies, Inc., Santa Clara, CA, USA). High-purity helium (6.0) served as carrier gas at a 1.0 mL/min constant flow rate. The GC oven temperature was programmed to increase from 60 °C (3 min isothermal) to 200 °C at 8ºC/min (2 min isothermal), from 200-230ºC at 10 ºC/min (5 min isothermal), and finally from 230–250 °C at 10 °C/min (1 min isothermal). Samples were introduced by direct injection (splitless); 1 μL (10 µL/mL sample in n-hexane) was injected at 0.7 mg/mL velocity with an Agilent 7683 autosampler. The injector temperature was 250 °C and the split ratio was 1:50 [60]. Samples were compared to analytical standards (Sigma Aldrich, Munich, Germany) in three technical replicates for the following marker molecules: cinnamaldehyde, cinnamyl acetate, eugenol, linalool, beta-caryophyllene, 1,8-cineole, trans-anethole, alpha-pinene, beta-pinene, carvacrol, para-cymene, para-mentha-1,4-dien. References for the active oily compounds and their presence in the selected plants and related species are collected in Additional File 4.

### Sodium dodecyl sulfate–polyacrylamide gel electrophoresis (SDS-PAGE) on salivary proteins

The effect of HD on salivary proteins was investigated by SDS-PAGE which was performed as described by Laemmli [61]. The separated protein bands were visualized using silver staining [62]. Unstimulated saliva was collected 8 h after eating and tooth brushing from healthy volunteers. The salivary protein investigation was the only experimental setup in this study that required ethical approval. The Regional and Local Research Ethical Committee approved this research under the registration number 5022—PTE 2015. This permission is valid until 31 January 2023. Immediately before collection, three times rinsing with sterile saline were performed. After centrifugation (5000 rpm, 5 min) the supernatant was homogenized and 400 µL of it was mixed with 400 µL of the mouthwash dilutions and other investigated substances for 60 s, as long as a normal rinsing maximally lasts. After another centrifugation step (13000 rpm, 5 min) 200 µL supernatant was separated for SDS-PAGE. 50 µL sample buffer (five times diluted) was added, then the mixture was boiled for 5 min at 100 °C. 10 µL processed sample was loaded to the gel. Gel concentration was set to 12.5%. As a marker, 10 µL bromophenol blue and low molecular weight marker set (Amersham Biosciences, Amersham, UK) was used. The running time was 45 min at constant 120 V, initial 20 mA, not exceeding 2W. The gel was fixed with glutaraldehyde and stained with colloidal silver. During the analysis, a calibration curve was created on the molecular weight markers using the relative migration distance (pixels) and log_10_ of the exact molecular masses (kDa).

### Cytotoxicity test by 3-(4,5-dimethylthiazol-2-yl)-2,5-diphenyltetrazolium bromide (MTT) assay

Cytotoxicity of the mouthwash was tested on human immortalized keratinocyte cell line, HaCaT (kindly provided by the laboratory of Prof. Tamás Bíró, Department of Immunology, Faculty of Medicine, University of Debrecen, Hungary). Cells were treated with the mouthwash in 96 well plates at 80–90% confluency with a dilution range of 1:4, 1:5, 1:6.67, 1:10 and 1:20 in complete Dulbecco's Modified Eagle Medium (DMEM, Merck, Burlington, MA, USA) for 60 s, along with untreated controls. All the cells were washed with Hanks’ buffer followed by the addition of 150 µL freshly prepared MTT stock in Hanks’ buffer, supplemented with glucose (Merck, Burlington, MA, USA) in each well. The treated plates were incubated for 4 h at 37 °C in 5% CO_2_. The MTT supernatant was removed and 100 µL dimethyl sulfoxide (DMSO, Merck, Burlington, MA, USA) was added to the cells followed by shaking on a plate shaker for 5 s. The absorbance of the dissolved formazan was measured at 570 nm on a Perkin Elmer EnSpire Multimode plate reader (Perkin Elmer, Waltham, MA, USA) in three technical replicates.

### Antioxidant capacity measurement by 2,2-Diphenyl-1-Picrylhydrazyl (DPPH) radical scavenging assay

Three different, popular, commercially available mouthwashes were selected, which were Listerine Freshburst (Johnson & Johnson, New Brunswick, NJ, USA), Elmex Sensitive (GABA International Holding GmbH, Münchenstein, Switzerland), and Cserszömörcés (Herbária Zrt, Budapest, Hungary) to compare our developed product with. The appropriate concentration of HD was determined as 4-times dilution of the original tincture based on the previous cytotoxicity and SDS-PAGE experiments. To assess the antioxidant capacity, a radical scavenging assay was performed on the mouthwashes, which is based on the absorbance decrease of the stable organic radical DPPH [33, 63]. In brief, 50 µL acetate buffer (100 mM, pH 5.5) followed by 50 µL blank / standard / mouthwash sample dilutions (250 mg/mL – 0.1 mg/mL) and finally 100 µL of 200 µM DPPH (dissolved in 96% ethanol, Sigma-Aldrich, Munich, Germany) were pipetted in 96-well microplates. After 60 min incubation in the dark at room temperature, changes in the absorbance at 517 nm were measured on a Perkin Elmer EnSpire Multimode plate reader (Perkin Elmer, Waltham, MA, USA). The results were compared to serial dilutions of Trolox (6-hydroxy-2,5,7,8-tetramethylchroman-2-carboxylic acid, Sigma-Aldrich, Munich, Germany) water soluble vitamin E standard. All experiments were done in three technical replicates.

### Antimicrobial activity tests

The tests were carried out with the most common oral pathogens, which have a proven role in caries, periodontal and inflammatory diseases, summarized in Table 3.

**Table 3:** Tested microorganisms and their role related to oral health

**Name**	**Reference number**	**Type**	**Occurrence**	**Characteristics**	Incubation conditions	Used media	Reference
*Bacillus subtilis*	SZMC 0209	Gram-positive	skin, oral flora, gastrointestinal tract	non-pathogen	30°C	Mueller–Hinton	[64, 65]
*Escherichia coli*	ATCC 25922	Gram-negative	oral flora, gastrointestinal tract	intestinal and extraintestinal pathogen	30°C	Mueller–Hinton	[66]
*Lactobacillus plantarum*	ATCC 10241	Gram-positive	oral flora	pH reducer	30°C	Rogosa	[6]
*Pseudomonas aeruginosa*	ATCC 27853	Gram-negative	oral flora	nosocomial pathogen	30°C	Mueller–Hinton	[67, 68]
*Staphylococcus aureus*	ATCC 29213	Gram-positive	oral flora	most common bacterial pathogen	30°C	Mueller–Hinton	[69]
*Streptococcus mutans*	ATCC 25175	Gram-positive	oral flora	pioneer bacteria of caries	30°C	Blood agar	[70]
*Streptococcus sanguinis*	ATCC 10556	Gram-positive	oral flora	pioneer bacteria of caries	30°C	Blood agar	[71]

The tested mouthwashes were Listerine Freshburst, Elmex Sensitive, Cserszömörcés and HD. Main active ingredients of Listerine Freshburst are thymol, eucalyptol, menthol, and methyl salicylate. Elmex Sensitive contains sodium fluoride, amine fluoride, levulinic acid, sodium levulinate as main compounds. Active ingredients in the Cserszömörcés are tannin extracts with high tannic acid content and thyme. Each mouthwash was used in the recommended concentrations written in the product sheet. The appropriate concentration of HD was determined as 4-times dilution of the original tincture based on the previous cytotoxicity and SDS-PAGE experiments.

#### Disc diffusion assay

To determine the antimicrobial activity of the mouthwashes, first we performed a disc diffusion assay. Adequate agar medium, such as Mueller–Hinton (Sigma-Aldrich, Munich, Germany), blood agar (Sigma-Aldrich, Munich, Germany), Sabouraud agar (Sigma-Aldrich, Munich, Germany), Rogosa agar (Sigma-Aldrich, Munich, Germany) was used with 1.8% agar concentration.

Our controls were 40 mg/mL Gentamicin (Sandoz, Basel, Switzerland), 2% chlorhexidine-digluconate (CHX, Molar Chemicals, Halásztelek, Hungary), and 10% ethanol (Molar Chemicals, Halásztelek, Hungary). Experiments were performed in five technical replicates.

10 µL of the microbial suspensions with 0.4 optical density (measured at 590 nm) were dispensed respectively on the agar surface on a 92 × 16 mm dish. Following 30 min incubation, a single sterile paper disc was placed on the agars loaded with 10 µL of the test samples. After 72 h incubation at 37 °C, the plates were scanned and digitally evaluated with a densitometry software using a Kodak Image Station 2000R (Kodak, Rochester, NY, USA). The largest diameter of inhibition zone was determined [72].

#### Resazurin assay

To detect microbial aerobic capability, the fluorogenic agent resazurin was used. The irreversible reaction of resazurin to resorufin depends on the reduced form of nicotinamide adenine dinucleotide phosphate (NADPH) or nicotinamide adenine dinucleotide (NADH) which are the reductants in the presence of the adequate enzymes [73].

First, the optimal OD for the microbial suspensions and the resazurin concentration were determined on a Hitachi F-4500 Fluorescence Spectrophotometer (Hitachi High-Technologies, Tokyo, Japan) using 2.5 mL sterile cuvettes (BrandTech, Essex, CT, USA).

Based on the results of the optimization step, a fluorescence assay was performed at 540 nm excitation and 590 nm emission wavelengths using a Biotek Synergy HT plate reader (Santa Clara, CA, USA), on 96-well flat bottom plates. Into 200 µL broth 10 µL bacteria sample plus 5 µL resazurin (final concentration of 5 µM) were added. The mixture was supplemented up to 250 µL with sterile saline solution. The fluorescence of 200 µL samples was measured after 60 min. 10% ethanol was used as control. Absolute control contained inoculated broth without resazurin to exclude background fluorescence changes. Tests were carried out in five technical replicates.

### Anti-Severe Acute Respiratory Syndrome Coronavirus 2 (SARS-CoV-2) assay

Before the anti-viral tests, toxicity of the mouthwash on Vero E6 cells was inspected using CellTiter-Glo® adenosine triphosphate (ATP) quantification kit (Promega, Madison, WI, USA). Briefly, the cells were incubated with the mouthwash for 30 min, then it was replaced by DMEM (Lonza, Basel, Switzerland) with 2% heat-inactivated FBS (Gibco, Waltham, MA, USA), and 1% Pen-strep (Lonza, Basel, Switzerland). Following a two-day long incubation CellTiter-Glo reagent was added to the cells and the luminescence signal was measured on a Perkin Elmer EnSpire Multimode plate reader (Perkin Elmer, Waltham, MA, USA) [74].

To examine the anti-SARS-CoV-2 activity of the HD mouthwash, Vero E6 cells were infected with a Hungarian SARS-CoV-2 isolate (GISAID ID: EPI_ISL_483637). The mouthwash was diluted in a 1:3 ratio in sterile distilled water, as it is determined by the previous experiments. For each experiment the mouthwash was diluted freshly. The 4-times diluted mouthwash was mixed in a 1:1 ratio with 0.3% bovine serum albumin (BSA) solution as an interfering substance [75–77]. 25 µl aliquots were mixed with 25 µl SARS- CoV-2 virus isolate (MOI: 0.01). After 1 min of exposure time, activity was neutralized by tenfold dilution in DMEM (Lonza, Basel, Switzerland), containing 2% heat-inactivated fetal bovine serum (FBS) (Gibco, Waltham, MA, USA) and 1% Pen-strep (Lonza, Basel, Switzerland). The total volume of the viral-oral wash solution was then added to Vero E6 cells. 30 min later the viral-oral wash solution was replaced by DMEM (Lonza, Basel, Switzerland) supplemented with 2% heat- inactivated FBS (Gibco, Waltham, MA, USA) and 1% penicillin–streptomycin (Lonza, Basel, Switzerland) [74]. Two days post infection, the cells were inspected under light microscope and ribonucleic acid (RNA) was extracted from the supernatant. Bio-Rad (Hercules, CA, USA) QX 200 Droplet- Digital PCR system was used to determine the viral copy number from the supernatant. The reaction was RNA-dependent RNA Polymerase (RdRp) gene specific, the primers and probes (Charité/Berlin assay) were ordered from Integrated DNA Technologies (Coralville, IA, USA). The RNA extracts were 100-times diluted prior to the droplet digital PCR. The experiments were done four times with 3 replicates.

### Analysis and statistics

During the analysis of the SDS-PAGE experiments equation of the linear regression was used to define the molecular weight of the selected bands after measuring their relative migration distance on the gel digital photograph.

The IC_50_ value (the dilution of the mouthwash in percentage needed to scavenge 50% of the DPPH) in the antioxidant capacity measurements for the tested mouthwashes were calculated by linear regression using the scavenging activities vs. amount of the mouthwash in each sample. The lower the IC_50_ value of the sample, the higher its antioxidant activity. The radical scavenging capacity of the mouthwash in percentage of the blank was calculated by the following equation:

$$\mathrm{Radical}\;\mathrm{scavenging}\;\mathrm{activity}\;\left(\%\;\mathrm i\mathrm n\mathrm h\mathrm i\mathrm b\mathrm i\mathrm t\mathrm i\mathrm o\mathrm n\right)=\left(\frac{{\mathrm A}_0-{\mathrm A}_1}{{\mathrm A}_0}\right)\times100$$

where A_0_ and A1 are absorbances of the blank and the samples, respectively [63].

100% dilution represented the recommended concentration of each mouthwash.

Pearson correlation and linear regression were used to assess the correlation between antioxidant capacity and antimicrobial activity.

One-way ANOVA and Dunnett’s post hoc test was used to compare whole lane densities in the SDS-PAGE experiments, to compare the results of the cytotoxicity assay, to analyze differences in the antioxidant capacity measurements, and to assess changes in viability of Vero E6 cells and copy numbers of SARS-CoV-2.

Two-way ANOVA with Dunnett’s post hoc test was used to compare band densities across the lanes in the SDS-PAGE experiments, to the statistical analysis in the disc diffusion assay, and to analyze the result of the resazurin assay.

Two-way ANOVA with Sidak's multiple comparisons test was used to compare the effectiveness of the mouthwashes against Gram positive and negative bacteria.

Level of significance was set at 0.05 for all the tests.

Integrated pixel density of the whole lanes and selected bands on the SDS-PAGE gel photograph were measured with Fiji (ImageJ2) software. Analysis and graphing were carried out in GraphPad Prism 8 (GraphPad Software, San Diego, CA, USA). Photoshop (Adobe Systems, San José, CA, USA) was used for creating figures and processing images.

## Results

### Market research

**Fig. 1:**
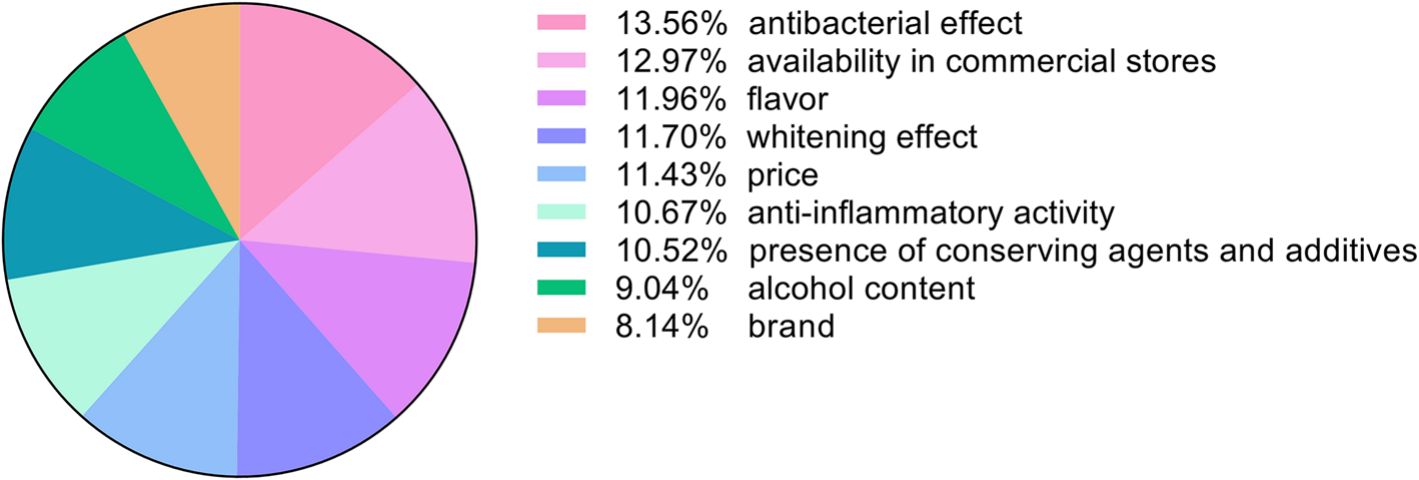
Parameters to identify the preferences of the customers on choosing a mouthwash. Importance of the listed parameters, scored on a scale from 1 to 6 (1 is the least important, 6 is the most important in choosing a mouthwash). Given scores were summarized and plotted for each parameter and presented as percentage distribution. Selection was based on our online survey and personal data collection (Additional File 1)

According to the openly available results on Statista Global Consumer Survey on the personal care product usage in Hungary, 90% of the responders use oral and dental care products regularly [26]. Our market survey was filled in by 381 volunteers (274 women, 108 man). More than half of the responders (60.70%) were between the age of 19–25. 54.86% of the total had university education or higher degree. 68.24% of the volunteers paid a visit not more than once a year at a dentist. Out of the total, 204 people were regular mouthwash users, who buy their favorite products mostly in hypermarkets and in cosmetic stores. The most popular product was Listerine: 44.12% of the regular users were applying it. 305 volunteers (80.05% of all responders) prefer the products from natural basis. All the regular users and 5 non-users stated that they would buy a mouthwash which has only natural ingredients and are even ready to visit herbal specialty stores to obtain it. The most important parameters based on which the responders choose the products are presented in Fig. 1.

Our online research on the ingredients included selected products from the Hungarian market. Researching the market supply, we found that all the rinses contain at least one synthetic compound, additive or preservative. The number of ingredients of the rinses’ ranges from 6 to 22 (Additional File 5).

### Quantitative analysis of the active compounds of the ingredients using GC/MS

**Fig. 2:**
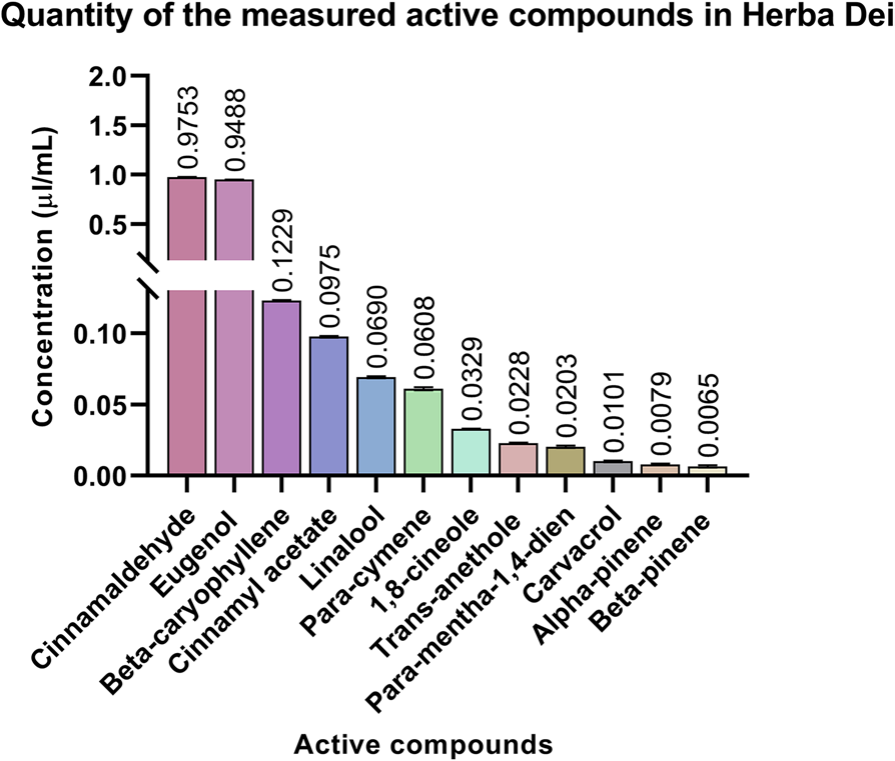
Amount of tested active compounds in our mouthwash. Bars represent the mean ± standard deviation (SD) of three technical replicates of the GC/MS measurements. Quantities are given in µl/mL

The quantitative assessment of the completely prepared mouthwash revealed that the main oily compounds responsible for biological effects are predominantly cinnamaldehyde and eugenol (Fig. 2).

### SDS-PAGE on salivary proteins

**Fig. 3:**
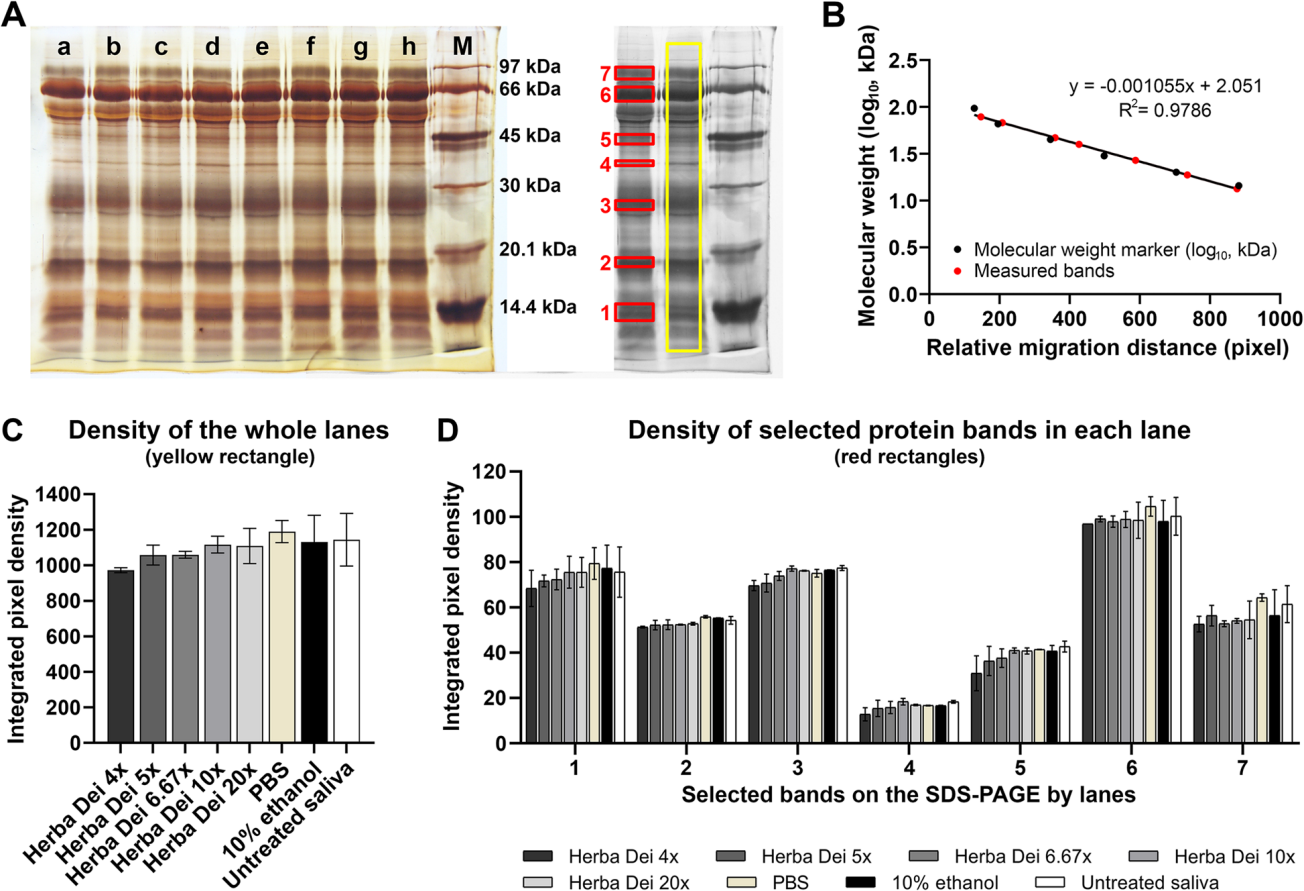
**A** Representative picture of the silver stained SDS polyacrylamide gel. Lanes represent the saliva samples mixed with the following substances: a. HD 4-times diluted, b. HD 5-times diluted, c. HD 6.67-times diluted, d. HD 10-times diluted, e. HD 20-times diluted, f. PBS, g. 10% ethanol, h. native saliva, M: low molecular weight protein marker. Yellow rectangles show the area in each lane the density was measured using Fiji (ImajeJ2) software. Numbered red rectangles show the bands selected for pixel intensity measurements in each lane to compare effects of different treatments. **B** Calibration of molecular weight based on the relative migration distance of the low molecular weight markers measured in pixels on the gel photograph. Black points show the molecular weight markers, whereas red points show the molecular weight (log_10_) of the selected bands based on their migration distance. y = -0.001055x + 2.051 is the equation of the linear regression, R^2^ = 0.9786 represents the goodness of fit, *p* = *0.0002*. **C** Integrated pixel density of the separate lanes on the SDS-PAGE (yellow rectangle in **A**). Bars represent the mean ± SEM of two technical replicates (two different gels loaded with the same samples). Ordinary one-way ANOVA with Dunnett’s multiple comparisons test. **D** Integrated pixel density of the selected bands (1 to 7, red rectangles in **A**) on the SDS-PAGE from each lane. Two-way ANOVA with Dunnett’s post hoc test, comparing every treatment to the native saliva. Bars represent the mean ± SEM of two technical replicates (two different gels loaded with the same samples)

Analyzing whole-lane densities (a-h on Fig. 3A), the SDS-PAGE experiments revealed that HD did not disturb the salivary protein integrity in either of the concentrations (Fig. 3C). Significant bands were also chosen to cover approximately all regions of the lanes in the low molecular weight range (1–7 in Fig. 3A). The investigated bands at the regions of 13–14 kDa (1), 18–19 kDa (2), 26–27 kDa (3), 39–40 kDa (4), 47–48 kDa (4), 54–55 kDa (5), 67–68 kDa (6), 78–79 kDa (7) did not show any statistical difference in their integrated pixel density compared to the native, untreated saliva (lane h) (Fig. 3A, D). Exact *p* values are listed in the Additional File 6. Original, uncropped gel photograph is presented in the Additional File 7.

### HD mouthwash showed minimal cytotoxicity to keratinocyte cells

**Fig. 4:**
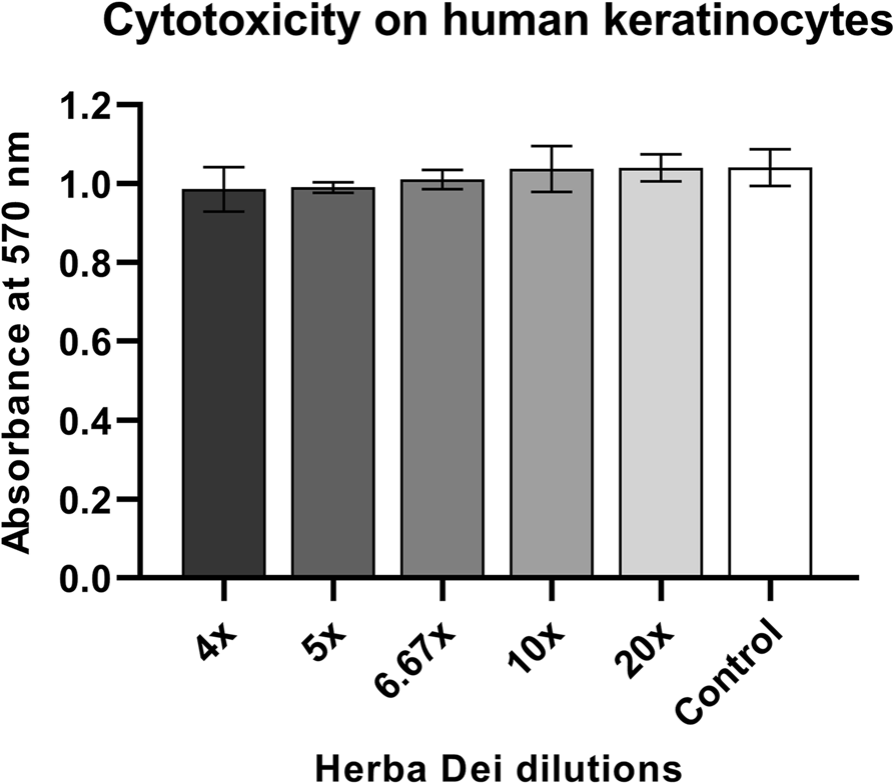
Cytotoxicity assay of different HD mouthwash concentrations. 4-times, 5-times, 6.67-times, 10-times and 20-times dilutions on HaCaT cell line in a 2D monolayer setting. Three technical replicates were performed. Ordinary one-way ANOVA with Dunnett’s multiple comparisons test. Bars represent the mean ± standard error of mean (SEM)

The viability of HaCaT cells did not show significant decrease following 60 s treatment with HD. All the concentrations were compared to the control, no statistical difference was found. At the 4-times dilution of the mouthwash, only a 5.32% decrease could be detected compared to the control samples (Fig. 4). *P* values are listed in the Additional File 6.

### Herbal-based mouthwashes show high antioxidant capacity

**Fig. 5:**
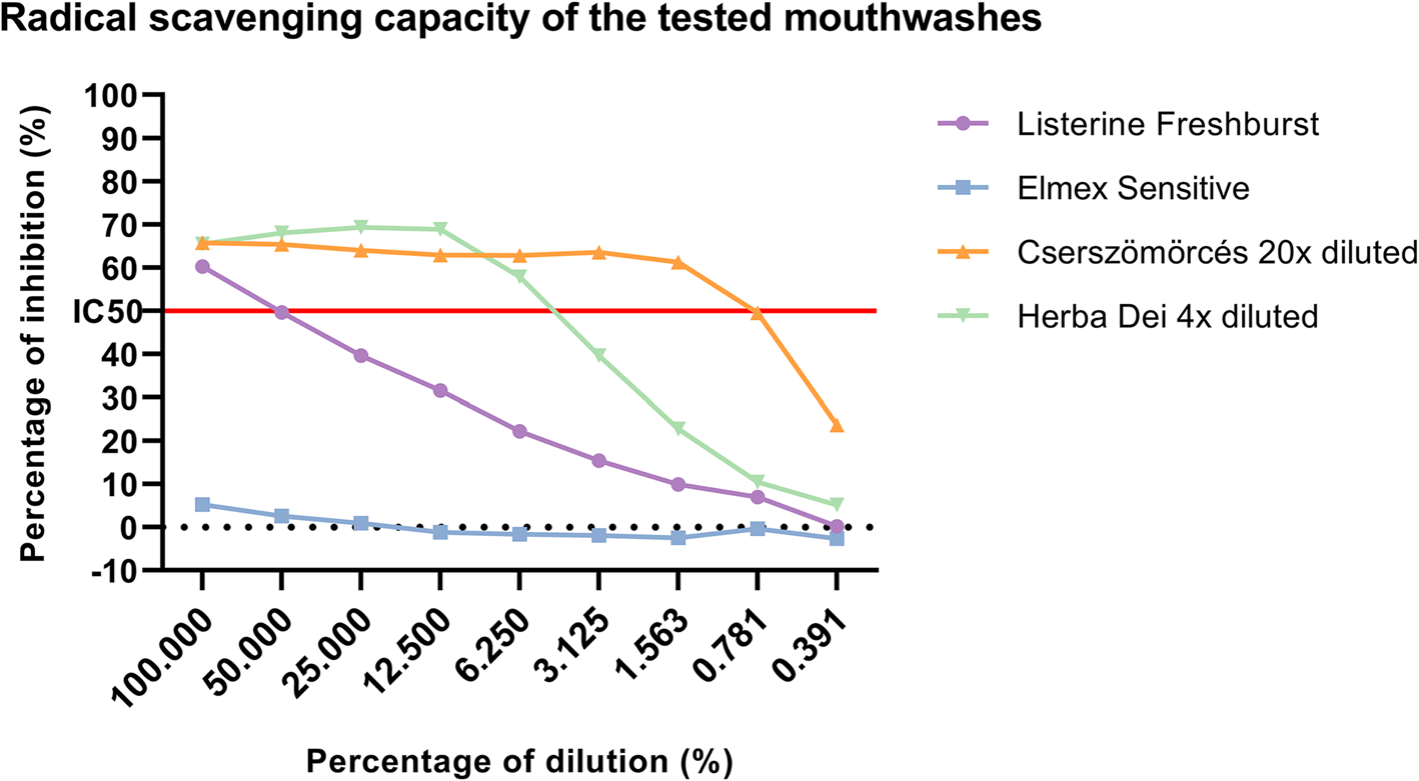
Antioxidant activity of the selected mouthwashes (Listerine Freshburst, Elmex Sensitive, Cserszömörcés, HD). 100% of dilution is equivalent with the recommended concentration for use of each product. Points represent the percentage of inhibition calculated from the mean of 3 technical replicates, red line shows the IC50 value

Out of the four tested mouthwashes Cserszömörcés exerted the highest radical scavenging capacity with an IC50 value at the dilution of 0.795%. HD showed the second highest antioxidant activity with the IC50 value at the dilution of 5.005%. Listerine Freshburst was less effective in scavenging radicals and had an IC50 value at 62.087%. Elmex Sensitive did not show any antioxidant activity in our experimental setup; IC50 value could not be interpreted in this case (Fig. 5).

### Antimicrobial activity

#### The antimicrobial activity of HD is comparable with that of the selected, commercially available mouthwashes in disc diffusion assay

HD in the recommended concentration showed lower antimicrobial activity against all the tested microbes compared to the positive controls, such as gentamicin (*p* < *0.0001*), and CHX 2% (*p* < *0.0001*) but had significantly higher effects than the 10% ethanol (*p* < *0.0001*), which is the solvent of the mouthwash.

HD turned out to be more effective than Listerine Freshburst against all the tested bacteria (*p* < *0.0001*), except for *Lactobacillus plantarum*, where Listerine Freshburst had the highest activity among all the tested mouthwashes.

HD showed similar effects without statistically significant difference as Elmex Sensitive when looking at *Streptococcus mutans* but had lower effect regarding *Bacillus subtilis* (*p* < *0.0001*), *Staphylococcus aureus* (*p* < *0.0001*), *Streptococcus sanguinis* (*p* < *0.0001*). At the samples of *Pseudomonas aeruginosa* (*p* < *0.0001*), *Escherichia coli* (*p* < *0.0001*) and *Lactobacillus plantarum* (*p* < *0.05*) HD was slightly more effective than Elmex Sensitive.

**Fig. 6:**
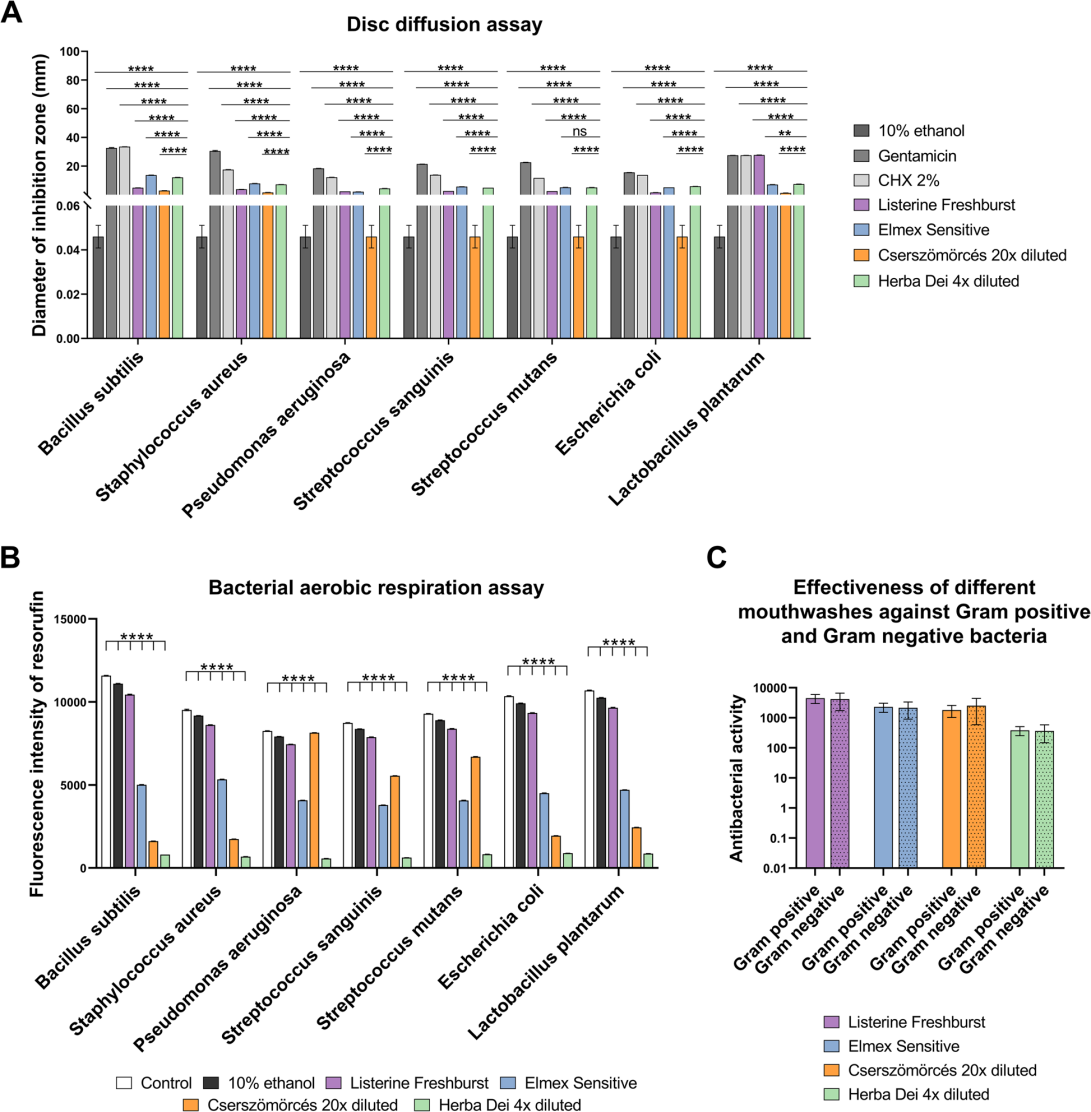
**A** Antimicrobial effects of Listerine Freshburst, Elmex Sensitive, Cserszömörcés, and HD in disc diffusion assay. Experiments were performed in five technical replicates on the adequate media with 1.8% agar concentration for each bacterium. Two-way ANOVA with Dunnett’s post-hoc test, comparing every agent to the HD 4-times diluted samples. Bars represent the mean ± SEM of the diameter of the inhibition zone in millimeter (mm). *p < *0.05*, **p < *0.01*, ***p < *0.001*, ****p < *0.0001*. **B** Aerobic respiration of the bacteria measured with resazurin assay following 60 s treatments with Listerine Freshburst, Elmex Sensitive, Cserszömörcés, and HD mouthwashes. 10% ethanol served as solvent control for HD. Control sample was untreated (media only). Two-way ANOVA with Dunnett’s post-hoc test, comparing every agent to the HD 4-times diluted samples. Bars represent the mean ± SEM of the fluorescence intensity of resorufin. *p < *0.05*, **p < *0.01*, ***p < *0.001*, ****p < *0.0001* **C**. Activity of the mouthwashes against Gram negative and positive bacteria. Y axis shows the cumulative results of the disc diffusion and resazurin assays. Two-way ANOVA with Sidak’s post hoc test

The activity of HD was significantly higher than that of the other herbal-based mouthwash, Cserszömörcés (*p* < *0.0001*) at all the investigated bacteria (Fig. 6A). Mean diameter of inhibition zones for each bacterium and tested mouthwash are listed in Table 4. *P* values are listed in detail in the Additional File 6.

**Table 4:** Mean diameter of inhibition zones for each bacterium and tested mouthwash

	10% ethanol	Gentamicin	CHX 2%	Listerine Freshburst	Elmex Sensitive	Cserszömörcés 20 × diluted	Herba Dei 4 × diluted
Bacillus subtilis	0.046	32.674	33.552	4.904	13.788	2.9	12.122
Staphylococcus aureus	0.046	30.562	17.59	3.798	7.912	1.652	7.188
Pseudomonas aeruginosa	0.046	18.428	12.228	2.36	2.102	0.046	4.464
Streptococcus sanguinis	0.046	21.424	13.88	2.646	5.684	0.046	4.922
Streptococcus mutans	0.046	22.618	11.72	2.534	5.218	0.046	5.132
Escherichia coli	0.046	15.586	13.832	1.588	5.192	0.046	5.912
Lactobacillus plantarum	0.046	27.536	27.536	27.732	7.17	1.318	7.488

#### The aerobic respiration of bacteria shows significant decrease following HD treatment in resazurin assay

The bacterial aerobic respiration measured in suspension based on the irreversible reaction of resazurin to resorufin following 30 min treatment with HD was significantly lower compared to all the tested mouthwashes, to 10% ethanol and to the control sample. *P* value is less than *0.0001* in all the comparisons (Fig. 6B).

#### All the tested mouthwashes are effective against both Gram positive and negative bacteria

No significant difference could be detected in the activity of the tested mouthwashes against Gram positive or negative bacteria (Fig. 6C). Details of the statistical test and *p* values are listed in Additional File 6.

### Antioxidant capacity of the mouthwashes did not correlate with their antibacterial activity

**Fig. 7:**
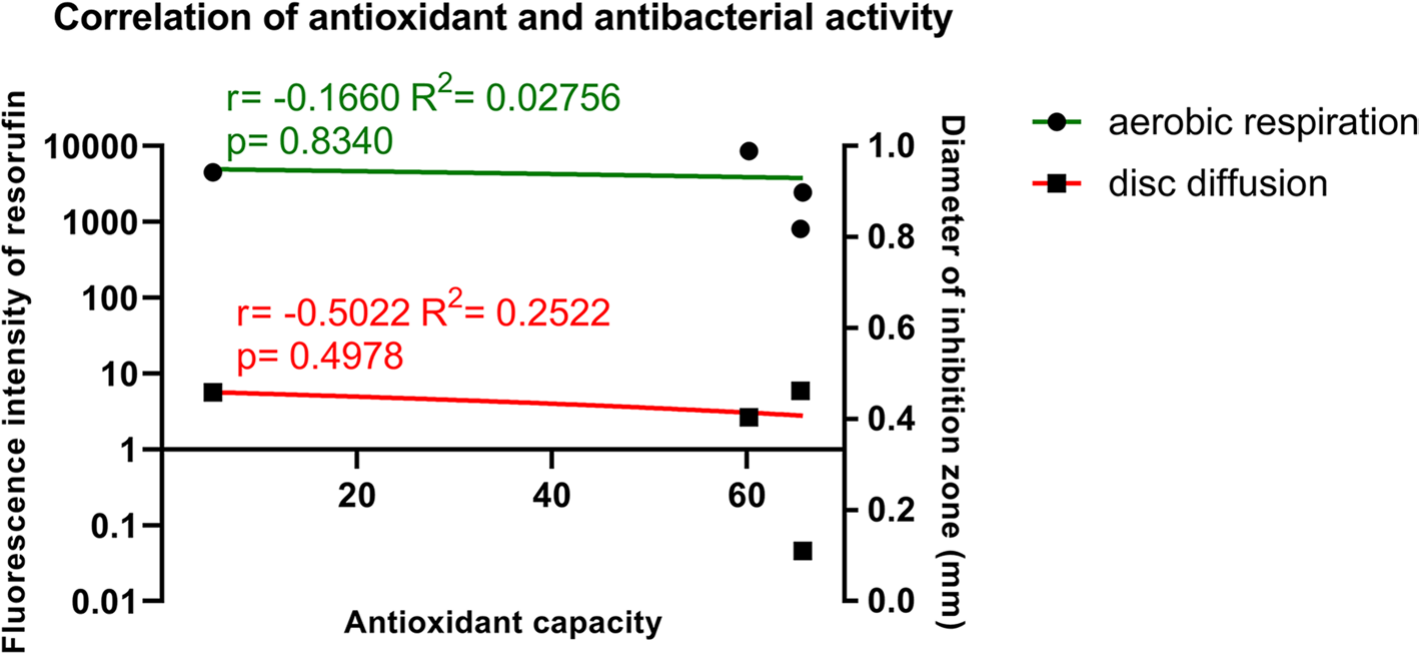
Correlation between the antioxidant capacity (x) and antibacterial activity of the tested mouthwashes. Cumulative results from all the microbes (mean values) were used for every mouthwash separately to compare to the results of the cell-free antioxidant capacity measurements. Points and green line represent the performance of the four tested mouthwashes in the bacterial aerobic respiration assay (corresponding y axis: left), squares and red line label their performance in disc diffusion assay (corresponding y axis: right). Pearson correlation and linear regression

Results of the resazurin and the disc diffusion assay were correlated to the antioxidant capacity of the different mouthwashes measured in the DPPH assay. No correlation could be revealed between the antioxidant and antibacterial activity of the tested mouthwashes (Fig. 7).

### HD mouthwash significantly lowered the SARS-CoV-2 copy number in vitro

A separate cytotoxicity test was performed on Vero E6 cells to assess the toxicity of the mouthwash exactly in the same conditions as the anti-SARS-CoV-2 experiments were carried out. HD in 4 × dilution itself and HD 4 × diluted + BSA did not lower the ATP content of the cells compared to the control.

**Fig. 8:**
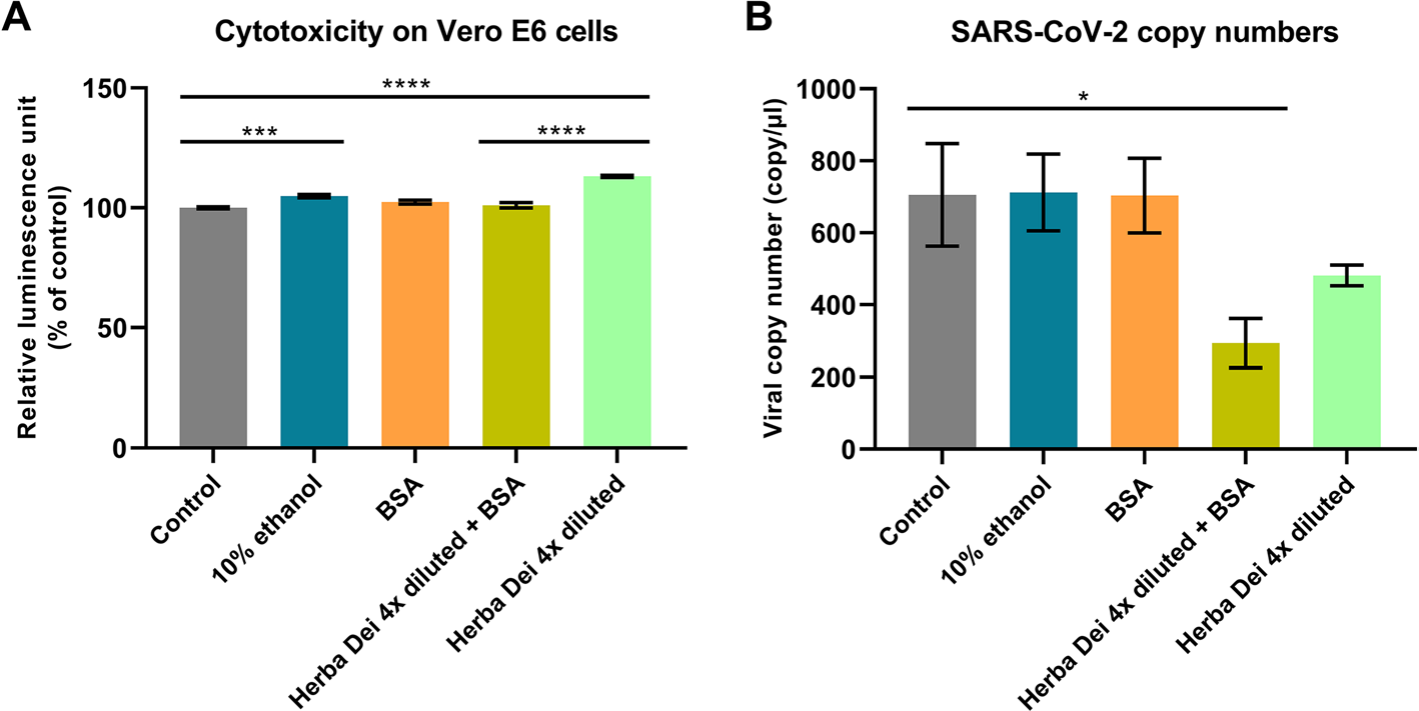
**A** Cytotoxicity test on Vero E6 cell line using the CellTiter-Glo assay. One-way ANOVA with Tukey’s post hoc test. Bars represent the mean ± SEM of the relative luminescence unit in % of control. **B** SARS-CoV-2 RdRp gene copy number alterations in the different treatments on Vero E6 cell line. Bars represent the viral copy number in copy/µl measured by droplet digital PCR. One-way ANOVA with Tukey’s post hoc test, mean ± SEM. *p < *0.05*, **p < *0.01*, ***p < *0.001*, ****p < *0.0001*

Inhibition of SARS-CoV-2 replication is well-marked at the HD 4 × diluted + BSA samples compared to the control (*p* < *0.0001*). Although the statistical probe did not show, the 4 × diluted HD itself (mean copy number: 481.5) also caused inhibition in the replication of the virus compared to the control (mean copy number: 705.5) and to the 10% ethanol (mean copy number: 712.2). This experiment also proved that it is not the 10% ethanol solvent of our mouthwash that might be responsible for the anti-viral effects, rather it is due to the herbal-based active compounds (Fig. 8).

## Discussion

In this work a novel, herbal-based mouthwash was created and evaluated using a combination of early pharmaceutical knowledge and modern chemical and biological testing methods. With this product, we aim to contribute to the oral healthcare market providing an entirely natural alternative to oral rinses and to contain the transmission of SARS-CoV-2. In the time of intensified science and vaccination denial [78], we hope to target those dental care patients with this mouthwash who oppose synthetic products, or even the SARS-CoV-2 vaccination.

The tight relationship between oral health, general well-being and life satisfaction is unquestionable. Oral health is not just an issue that can be approached from a subjective point of view but has exact economic and medical concerns in the western world, as well as in developing countries [79, 80]. Periodontal diseases and odontogenic infections can lead to secondary systemic consequences, such as cardiovascular problems, pneumonia, diabetes mellitus or chronic kidney disease, each of them exerting significant health-economic impact [81–83].

Herbal-based phytochemicals have gained an advantage in recent years in oral health care which is not only a result of the need for economic solutions, especially in developing countries, but also because they are an effective alternative to the problem of the multidrug-resistant pathogens and to the side effects of the synthetic products [25, 84]. Another important aspect is the environmental load of the cosmetics and healthcare solutions both at the level of manufacturing [85] and at the end-user [86]. Natural, local, renewable, and herbal-based components offer an alternative in case the industry faces challenges because of the lack of raw materials [87]. To support and establish sustainability and reduce water pollution it is wise to choose products based on their low carbon footprint and biodegradability.

In the recent decades, a concern has been raised about toxic chemicals in our body originating from cosmetics [88]. Considering the tendency of health care product usage nowadays, we apply approximately up to 100 chemicals a day. Natural substances in this regard are less likely to have side effects [89]. For example, essential oils are “Generally Recognized as Safe” (GRAS) by the United States Food and Drug Administration (FDA) to be used in food and cosmetics by experts without safety issues [33, 90].

Besides that HD is biodegradable, the toxicity and salivary protein integrity experiments showed that the toxic load exerted by the mouthwash in the oral cavity is very low. Moreover, considering the ethanol content of HD, which serves as a solvent for the non-polar compounds, a recent systemic review and meta-analysis revealed that there is no sufficient evidence to support the proposition that ethanol containing mouthwashes contribute to the development of oral cancer [91].

In case of oral care products, the importance of keeping the integrity of the oral protein environment must be emphasised, because the saliva contains proteins which are important parts of the local digestive and defence system. Selectively or entirely disrupting these factors every day can lead to just as serious consequences as the untreated periodontal diseases [92, 93]. Although, proteins were not defined by using antibodies, pixel density was measured in the SDS-PAGE experiments at regions which are reported to be around or near to the molecular weight of the major salivary proteins, such as lactoferrin and lactoperoxidases at 78–80 kDa [94, 95], amylase isoforms between 55–65 kDa [96, 97], defensins around 40–45 kDa [98], proline-rich protein isoforms in three or more major bands from 14 to 40 kDa [99], lysozyme, cathelicidin from 15 to 18 kDa [100, 101], salivary cystatins [102] and very low molecular weight proline-rich proteins [99] at 13–15 kDa. Our product performed very well at this key point, proving that efficient antibacterial activity can be reached using natural ingredients as well, without disrupting the oral environment the mouthwash must work in.

Oral care includes the treatment of any wounds that may occur in the oral cavity. A mouthwash besides the cleaning effect should facilitate the contraction and healing of wounds by mediating inflammation through antioxidant capacity [103]. Moreover, antioxidants can also compensate for the local irritative agents like smoke, can help reduce halitosis [104], and are as effective in reducing bleeding as CHX solutions [105]. Including herbal-based compounds with proven radical scavenging activity could be a possibility in this regard. Phenylpropanoids, terpenes, and terpenoids are present in significant quantities in plant extracts used to create our mouthwash and are already proven to have great potential in preventing oxidative damage [106, 107]. Bitter components, like tannins, and flavonoids in our product can reduce the amount of reactive oxygen species and induce angioneogenesis and epithelial cell proliferation, promoting wound healing [63]. The anaesthetic effect must be mentioned as well, which is a primary requirement in the treatment of oral lesions, because of the rich innervation of the mouth.

The accumulation of microbial flora is responsible for plaque and biofilm formation on the oral surfaces [5, 6]. The capability of herbal-based extracts and their active components to inhibit the growth of these microorganism populations have been previously published [108, 109]. Our natural mouthwash formulation was found to be rich in terpenoids which include carvacrol, cineol, cymene, eugenol, linalool, and pinene. Previously reported data suggest that the bacterial cell wall and membrane disruption by these molecules along with ATP and ion leakage occur due to diversely positioned hydroxy groups around the phenolic rings of the terpenoids, which might be one of the major causes for the antimicrobial property of the herbal-based oily compounds [110, 111]. Cinnamaldehyde is proven to cause membrane disruption by forming lipid raft aggregates [112]. Eugenol has dose-dependent inhibiting effect on bacterial growth in the log phase, and on ATP generation [113]. β-caryophyllene is reported to inhibit bacterial efflux pumps [114], and to cause non-selective pore formation in bacterial membrane [115]. Linalool interferes with the tricarboxylic acid cycle and glycolysis pathway [116]. Trans-anethole was efficient in increasing susceptibility to other anti-microbial agents, and in hindering biofilm formation thus, proved to be able to protect against *S. aureus* recolonization [117]. Carvacrol had effects on bacterial membrane ion gradients, even without causing significant ATP leakage [110]. Alpha-pinene induced apoptosis by increasing the caspase-3 activity [118], but had significant synergistic interactions as well, which might be promising to various therapeutic fields [119].

The microbial susceptibility analysis done on the tested mouthwashes showed that our plant extract and essential oil-based natural mouthwash formulation successfully inhibited the growth of the usual Gram-positive and negative oral bacteria, which is consistent with previous studies made on herbal-based products [120–122]. Although the growth inhibition by HD was significant, it was also less robust than a treatment with antibiotics or CHX, which is not a drawback, because in everyday use it is not beneficial to completely eradicate the oral flora.

The significance of oral care regarding respiratory virus infections and transmission has been proven in the past few years during the COVID-19 pandemic more than ever [123]. Oral health care products act at the front sites when it comes to upper respiratory pathogens thus, their importance in adding a level to our defence came to the focus recently [77, 124]. HD proved to be effective in lowering the viral copy number of SARS-CoV-2 under in vitro conditions, anticipating the possibility that it could reduce the viral load of saliva, contributing to infection control not only in the everyday interpersonal contact, but under aerosol-generating conditions, such as dental treatments, achieving all this in a natural way. Our mouthwash was performing best in the anti-viral assay at conditions which are mimicking the intraoral environment thus, are the closest to the intraoral usage. As for the treatment of COVID-19 related oral complications, the most important features of a mouthwash are the anti-viral, antibacterial, and anti-inflammatory activity. Since SARS-CoV-2 might bind to salivary mucins, and to the angiotensin-converting enzyme 2 (ACE2) receptor on the tongue, resulting in the distortion of gustatory sensation [123, 125], reducing the viral load of the saliva has a direct beneficial effect on the development of dysgeusia and oral lesions. In most cases a coinfection is present in the oral lesions and no bacterial sensitivity is provided to support treatment [126] thus, it might be beneficial to use a product which has an acceptable efficiency against a wide spectrum of Gram positive and negative bacteria but does not eradicate the oral flora entirely.

Our results support the idea of using herbal-based antioxidant, anti-bacterial and anti-viral components as the major elements of mouthwashes and oral cleansers. Moreover, these components based on their anti-halitotic and flavouring effects, have the potential to be applied as natural active ingredients in mouthwashes, making the use of additional flavouring and preservatives unnecessary [127]. Following the licencing procedure and market appearance, our product gained a significant popularity among local oral health specialists and patients who were looking for natural alternatives which are experimentally evaluated. Local manufacturing ensures a cooperative control over production, as a beneficial consequence of which wasting could be reduced to zero.

## Conclusions

Our product proved to be effective against the most common oral bacteria without disrupting the oral salivary environment, and in reducing SARS-CoV-2 copy number in vitro. Moreover, its antioxidant activity might help in healing wounds and to compensate for irritative agents.

## Supplementary Information

**Additional file 1.** Questions of the market survey – translated. Translated questionnaire of our online market survey.

**Additional file 2.** Herbal material quality assurance certificates. Description of data: International Organization for Standardization (ISO) 9001 and International Featured Standards (IFS) qualification of the herbal material provider.

**Additional file 3.** Data sheet on quality assurance of the essential oils. Quality information and safety data sheet of the essential oils.

**Additional file 4.** Summary table and references for the active oily compounds and their presence in the selectedplants and related species. A summary table, references and PubChem CID of the active oily compounds and their presence in the selected plants and related species.

**Additional file 5.** Ingredients of different mouthwashes, categorized by the EWG numbers. Ingredients of different mouthwashes, categorized by the EWG numbers. *This ingredient’s score is higher if used in products intended for use around mouth and on lips due to increased risk of ingestion and absorption. †This ingredient’s score is higher if used in inhalable products (e.g., sprays, powders) because of respiratory concerns. ‡This ingredient’s score is higher if used in products intended for use around mouth, on lips, around the eyes and for damaged skin due to increased risk of ingestion and absorption [23].

**Additional file 6.** Detailed statistical results and *p* values for the SDS-PAGE, cytotoxicity and disc diffusion. assays.

**Additional file 7. **Original image of the silver stained SDS polyacrylamide gel for Fig. 2.

## Data Availability

Data generated or analyzed during this study are included in this published article and its supplementary information files, except for the raw data of the market survey questionnaire, which is available upon reasonable request from the corresponding author.

## Abbreviations

CPNP: Commission cosmetic products notification portal
EWG: Environmental Working Group
GC/MS: Gas chromatography/mass spectrometry
SDS-PAGE: Sodium dodecyl sulfate–polyacrylamide gel electrophoresis
MTT: 3-(4,5-Dimethylthiazol-2-yl)-2,5-diphenyltetrazolium bromide
HaCaT: Human immortalized keratinocyte cell line
DMEM: Dulbecco's Modified Eagle Medium
DMSO: Dimethyl sulfoxide
DPPH: 2,2-Diphenyl-1-Picrylhydrazyl
CHX: Chlorhexidine-digluconate
NADPH: Reduced form of nicotinamide adenine dinucleotide phosphate
NADH: Reduced form of nicotinamide adenine dinucleotide
ATP: Adenosine triphosphate
SARS-CoV-2: Severe Acute Respiratory Syndrome Coronavirus 2
BSA: Bovine serum albumin
FBS: Fetal bovine serum
RNA: Ribonucleic acid
FDA: United States Food and Drug Administration
