# Supplementary material for: Development of a novel, entirely herbal-based mouthwash effective against common oral bacteria and SARS-CoV-2
Source: BMC Complement Med Ther. 2023 May 1;23:138. doi: 10.1186/s12906-023-03956-3 (PMC10150350; doi:10.1186/s12906-023-03956-3)
Supplement: Supplementary file 1 — Additional file 1. Questions of the market survey – translated. Translated questionnaire of our online market survey. [file 12906_2023_3956_MOESM1_ESM.docx]

Questions of the online market survey on mouthwash usage habits

1. Are you a regular mouthwash user?
2. If yes, which brand(s) are you using?
3. If not, then what is the reason?
4. If yes, how often are you using mouthwash?
5. How important is the price of mouthwash to you?
6. How important is the brand of mouthwash to you?
7. How important is the taste of mouthwash to you?
8. How important is the additive and preservative content of a mouthwash to you?
9. How important is the alcohol content of a mouthwash to you?
10. How important is the antibacterial effect of a mouthwash to you?
11. How important is the whitening effect of a mouthwash to you?
12. How important is the availability of a mouthwash to you?
13. How important is the anti-inflammatory effect of a mouthwash to you?
14. What taste of mouthwash do you enjoy?
15. How much do you spend on mouthwash per month?
16. Do you prefer products based on natural ingredients?
17. How much would you be willing to pay per month for an exclusively natural mouthwash?
18. Where do you buy mouthwash?
19. Do you usually shop at herbal specialty stores?
20. Would you be willing to go to an herbal store for a mouthwash with only a natural ingredient?
21. Have you ever bought an oral care product from your dentist?
22. How often do you go to the dentist?
23. Would you buy natural mouthwash from your dentist?
24. What is your gender?
25. What is your age?
26. What is your highest educational degree?
27. Which county are you living in?
