# Supplementary material for: Development of a novel, entirely herbal-based mouthwash effective against common oral bacteria and SARS-CoV-2
Source: BMC Complement Med Ther. 2023 May 1;23:138. doi: 10.1186/s12906-023-03956-3 (PMC10150350; doi:10.1186/s12906-023-03956-3)
Supplement: Supplementary file 2 — Additional file 2. Herbal material quality assurance certificates. Description of data: International Organization for Standardization (ISO) 9001 and International Featured Standards (IFS) qualification of the herbal material provider. [file 12906_2023_3956_MOESM2_ESM.pdf]

Tanúsítvány

Szabvány **ISO 9001:2008**

Tanúsítvány jegyzéksz. **75 100 8126**

Tanúsítvány birtokosa: **HERBÁRIA Gyógynövény- feldolgozó és Kereskedelmi Zrt.**
Csata u. 27.
H - 1135 Budapest
Magyarország

A mellékletben felsorolt telephelyekkel.

Alkalmazási terület: gyógy- és fűszernövények feldolgozása és értékesítése, gyógynövény tartalmú készítmények fejlesztése, gyártása és értékesítése

tevékenységi területén működtetett menedzsment rendszere megfelel az MSZ EN ISO 9001:2009 (ISO 9001:2008) szabvány követelményeinek.

Érvényesség időtartama: A tanúsítvány érvényes **2017.12.03**-tól **2018.06.04**-ig.

Budapest, 2017.12.03.

TÜV Rheinland InterCert Kft.
H-1132 Budapest, Váci út 48/a-b
www.tuv.hu

NAH-4-0054/2015
IRÁNYÍTÁSI RENDSZEREKET TANÚSÍTÓ SZERVEZET

TÜVRheinland®
Precisely Right.

Bureau Veritas Certification

BUREAU VERITAS 1828

Certificate

Herewith the certification body

Bureau Veritas Polska Sp. z o. o.

Bureau Veritas Polska Sp. z o. o. being an ISO/IEC 17065 accredited certification body for IFS certification and having signed an agreement with the IFS Management GmbH, confirms that the processing activities of the above organisation meet the requirements set out in the standard

Awarded to

HERBÁRIA ZRT

Kamilla 2. 6821 Székkutas, Hungary

COID: 49826
GS1 GLN(s): 5990662914003
Sanitary Legal Authorization No: HU-10815678

IFS Food
Version 7, October 2020
and other associated normative documents

at **Higher Level**
with a score of 96,96%

for the Assessment scope:

Processing of herbs by drying, conditioning with water right before cutting, mixing, cleaning and milling packed in paper filter bag and paper bag. Production of tea products in paper filter bag and paper bag.

Exclusion: packaging of soft capsule into plastic boxes from bulk and packaging of Himalaya salt into 1000g bag

Beside own production, company has partly outsourced processes and/or products.

The company has own broker activities which are not IFS Broker/other GFSI recognized standard certified.

Product Scope: **10. Dry products, other ingredients and supplements**

Technology Scope: **C, E, F**

Assessment Date: **23-24.06.2022**

Date of the last unannounced Assessment: **NA**

Date of expiration of the certificate: **31.08.2023**

Next assessment to be performed within the time period:

between 12.05.2023 and 21.07.2023 in case of announced Assessment

between 17.03.2023 and 21.07.2023 in case of unannounced Assessment

Certificate- Register number: **INT2022324PL**

Date of issue of the certificate: Warsaw, **18.08.2022**

PCA POLSKIE CENTRUM AKREDYTACJI CERTYFIKACJA WYROBÓW AC 182

IFS Food

Witold Dżugan – Certification Manager

Managing Office:
Bureau Veritas Polska Sp. z o.o.
ul. Migdałowa 4 · 02-796 Warszawa · Poland
