# Supplementary material for: Development of a novel, entirely herbal-based mouthwash effective against common oral bacteria and SARS-CoV-2
Source: BMC Complement Med Ther. 2023 May 1;23:138. doi: 10.1186/s12906-023-03956-3 (PMC10150350; doi:10.1186/s12906-023-03956-3)
Supplement: Supplementary file 3 — Additional file 3. Data sheet on quality assurance of the essential oils. Quality information and safety data sheet of the essential oils. [file 12906_2023_3956_MOESM3_ESM.pdf]

Kiadás száma:	1.2
Kiadás kelte:	2012.11.22.
Felülvizsgálat kelte:	2013.04.19.

1. AZONOSÍTÁS

Anyag/készítmény megnevezése:

- magyar név: Fahéj olaj
- angol név: Cinnamon oil
- INCI:

Felhasználási területek: Illatszerek, kozmetika
CAS-szám:
EINECS:
Gyártó/forgalmazó adatai:

- Cégnév: Aromax Zrt.
- Cím: 1031 Budapest Záhony utca 7
- Tel: +36 1 8808 480 Fax: +36 1 8808 481 E-mail: aromax@aromax.hu

Egészségügyi Toxikológiai Tájékoztató Szolgálat: 1096 Budapest, Nagyvárad tér 2.
Tel: +36 80 201 199 Fax: +36 1 476-1138

2. VESZÉLYEK AZONOSÍTÁSA

Az anyag vagy keverék osztályozása:

Az 1272/2008/EK rendelet szerinti osztályozás
Acute Tox 4 H312 Bőrrel érintkezve ártalmas
Eye Irrit 2 H319 Súlyos szemirritációt okoz
Skin Irrit. 2 H315 Bőrirritáló hatású
Skin Sens. 1 H317 Allergiás bőrreakciót válthat ki.
Aqutic Chronic 3 H412 Ártalmas a vízi élővilágra, hosszan tartó károsodást okoz.

A 67/548/EGK irányelv vagya 1999/45/EK irányelv szerinti osztályozás
Xn, Ártalmas
R21 Bőrrel érintkezve ártalmas
Xi. Túlérzékenységet okozó
R38 Bőrizgató hatású
R43 Bőrrel érintkezve túlérzékenységet okozhat (szenzibiláló hatású lehet)
N; Környezeti veszély
R52/53 Ártalmas a vízi szervezetekre, a vízi környezetben hosszan tartó károsodást okozhat
Az emberek és a környezet veszélyeztetettségére vonatkozó különleges információk
A termék jelölése a többször módosított „Készítmények általános besorolási EK-irányelvei” számítási eljárása értelmében kötelező

Aromax Zrt.

Osztályozási rendszer:
Az osztálybasorolás megfelel az érvényes EK listáknak, de kiegészítik a szakirodalomból származó és a cégek által megadott adatok.

Címkézési elemek:
Az 1272/2008/EK rendelet szerinti cimkézés
A termék a CLP-rendelet (Anyagok és Keverékek Osztályozásáról, Cimkézéséről és Csomagolásáról szóló rendelet) szerint nincs osztályozva és címkézve

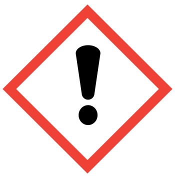

Figyelmeztető veszély
Veszélyt megahtározó komponensek a cimkézéshez
Fahéjaldehid
Eugenol
Trans-anethole
DIPENTENE

Veszélyességi intézkedés(ek):

H317 Allergiás bőrreakciót válthat ki
H312 Bőrrel érintkezve álrtalmas
H319 Súlyos szemirritációt okoz
H412 Ártalmas a vízi élővilágra, hosszan tartó károsodást okoz
H315 Bőrirritáló hatású

Elővigyázatossági intézkedések:

P261 Kerülje a por/füst/gáz/köd/gőzök/permet belélegzését
P280 Védőkesztyű/védőruha/szemvédő/arcvédő használata kötelező
P305+P351+P338 Szembe kerülés esetén: Több percig tartó óvatos öblítés vízzel. Adott esetben a kontaklencsék eltávolítása, ha könnyen megoldható. Az öblítés folytatása
P321 Szakellátás (lásd a címkén)
P322 Különleges intézkedések (lásd a címkén)
P501 Rendelkezzen a tartalomról/tárolásról a helyi/regionális/nemzeti/nemzetközi előírásoknak megfelelően

Egyéb veszélyek:
A PBT és vPvB értékelés eredményei

Aromax ZRt.

PBT: Nem alkalmazható
vPvB: Nem alkalmazható

3. ÖSSZETÉTELRE VONATKOZÓ INFORMÁCIÓK

Kémiai jellemzés:Keverékek

**Kémiai név**	**Tartalom (%)**	**CAS szám**	**Veszélyesség***	**Osztályozás (Direktíva 67/548/EEC)***
Fahéjaldehid	50-100%	104-55-2	Acute Tox. 4, H312; Skin Irrit 2. H315; Eye Irrit 2, H319; Skin Sens 1A, H317	Xn R21; Xi R38; Xi R43
Cinnamyl acetate	5-9,99	103-54-8	H316	
Linalool	2,5-5	78-70-6	Skin Irrit. 2, H315; Eye Irrit 2, H319	Xi R38
Eugenol	2,5-5	97-53-0	Eye Irrit. 2, H319; Skin Sens 1. H317	Xi R36; Xi R43
Beta-Caryophyllene	1-2,49	87-44-5	Asp.Tox. 1, H304	Xn R65
1,8 Cineol	1-2,49	470-82-6	H226; Flam.Liq 3	R10
Trans-Anethole	1-2,49	4180-23-8	Skin Sens 1, H317	Xi R43
DIPENTENE	1-2,49	138-86-3	Flam. Liq 3, H226; Asp. Tox. 1; H304; Aquatic Acute 1, H400; Aquatic Chronic 1, H410; Skin Irrit 2. H315; Skin Sens. 1, H317	Xn R65; Xi R38; Xi R43; N R50/53
p-Menth-1-en-8-ol	1-2,49	98-55-5	Skin Irrit. 2, H315; Eye Irrit. R, H319	Xi R38
p-Cymene	1-2,49	99-87-6	Flam Liq. 3, H226; Asp. Tox1, H314; Aquatic Chronic 2, H411	R10
Alpha Pinene	<1	7785-26-4	Flam. Liq 3, H226; Asp. Tox. 1; H304; Skin Irrit. 2, H315; Skin Sens. 1, H317	Xn R65; Xi R43; R10;
Benzil-benzoát	<1	120-51-4	Aquatic Chronic 2; H411; Acute Tox 4; H302	Xn R22; N R51/53
Beta-Pinene	<1	18172-67-3	Flam. Liq 3, H226; Asp. Tox. 1; H304; Skin Irrit. 2, H315; Skin Sens. 1, H317	Xn R65; Xi R38; Xi R43

*A termékre vonatkozó veszélyességi utalások szövege a 16. fejezetben található.

4. ELSŐSEGÉLYNYÚJTÁSI INTÉZKEDÉSEK

4.1. A szükséges elsősegélynyújtási intézkedések

Belégzés esetén:	A sérültet friss levegőre kell vinni. Ha nem lélegzik, mesterséges lélegeztetést kell alkalmazni. Nehézlégzés esetén adjunk oxigént. Azonnal hívjunk orvost.
Bőrrel való érintkezés esetén:	A szennyezett ruházatot távolítsuk el, a szennyeződött bőrfelületet bő vízzel mossuk le. Ha a tünetek fennmaradnak, hívjunk orvost.
Szembe kerülés esetén:	Öblítsük bő langyosvízzel legalább 15 percen keresztül, a szemhéjakat szélesre nyitva. Hívjunk szemorvost.
Lenyelés esetén:	Öblítsük ki a szánkat hidegvízzel, és forduljunk orvoshoz. Maradjunk nyugalmi helyzetben, ne próbáljunk hányni.

4.2. A legfontosabb akut és krónikus tünetek és hatások

Nem áll rendelkezésre információ.

4.3. Bármely azonnal szükséges orvosi odafigyelés és speciális kezelés

Nem áll rendelkezésre információ.

5. TŰZVÉDELMI INTÉZKEDÉSEK

5.1. Oltóközeg

Víz spray, széndioxid, száraz kémiai por vagy megfelelő hab

Biztonságtechnikai okokból ne használjunk teljes vízsugarat.

5.2. Vegyület vagy keverék használatából adódó speciális kockázatok

Ismert módon vagy várhatóan veszélyes éghető termékek: égés során toxikus füstöt bocsátanak ki.

5.3. Tűzoltási tanácsok

Egyéb tűzoltási megfontolások: a magas hőmérséklet zárt tartályokon belüli magas nyomáshoz vezethet.

Kerüljük a keletkező gőzök belélegzését. Használjunk légzőkészüléket. A tűz ne terjedhessen szennyvízcsatornákba és vízfolyásokba.

Speciális tűzvédelmi eljárások: viseljünk önhordó légzőkészüléket és védőruhát, hogy megelőzhessük a bőrrel és szemmel való érintkezést.

.

Aromax Zrt.

6. ÓVINTÉZKEDÉSEK BALESET (VÉLETLEN KIÖMLÉS) ESETÉRE

6.1. Személyes elővigyázatosság, védőberendezések és sürgősségi eljárások
Viseljen légzőkészüléket, kémiai védőszemüveget, gumicsizmát, és keménygumikesztyűt. Ne engedjen be másokat a kiürített területre. Kerülje el a gőzök belélegzését, használjon megfelelő légzőkészüléket.

6.2. Környezeti óvintézkedések

Hogy megelőzzük a környezet esetleges elszennyeződését, ne hagyjuk, hogy az anyag szennyvízcsatornába, felszíni vizekbe vagy a talajvízbe jusson.

6.3. Közömbösítő és takarítószerek és -módszerek

Szórjuk be inert, szervetlen, nem gyúlékony abszorbens anyaggal (pl. mészkő, homok, nátriumkarbonát).

Nem feltöltődő eszközt használva tegyük fedett konténerekbe, és szállítsuk el. Kerüljük a nyílt láng és gyújtóforrások (pl. a vízmelegítő őrlángja) használatát. Szellőztessük a területet, és mossuk fel a kiömlési területet, miután teljesen eltávolítottuk az anyagot.

Intézkedjünk az érvényes törvényekkel és szabályzatokkal összhangban.

6.4. Más pontokra hivatkozás

Nem értelmezhető.

7. KEZELÉS ÉS TÁROLÁS

7.1. Biztonságos kezelési óvintézkedések

Gondoskodjunk a munkaterület megfelelő szellőztetéséről, mert a gőzök robbanásveszélyes elegyet alkothatnak a levegővel.

Kerüljük el a szemmel, bőrrel és ruházattal való érintkezést. Viseljünk védőruhát és használjunk védőkesztyűt.

Tartsuk be a munkahigiéniai és –biztonsági előírásokat.

Csak eredeti konténerben tároljunk.

7.2. A biztonságos tárolás szabályai, beleértve a nem megfelelőséget

Ajánlott csomagolóanyagok: szorosan zárt és lehetőleg tele konténerekben, hűvös, száraz és szellőztetett területen tárolandó

Tároljuk üveg, megfelelő műanyag, alumínium vagy zománcozott konténerekben

Tárolás: tartsuk az anyagot gyújtóforrástól távol (pl. forró felszín, szikra, láng és statikus feltöltődés)

Védjük a hő/túlhevülési- és fényforrásoktól.

Tartsuk távol a nem megfelelő anyagoktól (lásd a nem megfelelőségi részt)

Akadályozzuk meg az illetéktelen személyek hozzáférését

Ne tároljuk az anyagot élelmiszer és ivóvíz közelében.

Ne nyissuk ki a konténert nyomás alatt.

7.3. Speciális használat

Használati óvintézkedések: a munkaterületen tilos a dohányzás.

Aromax Zrt.

8. EXPOZÍCIÓ ELLENŐRZÉSE / SZEMÉLYI VÉDELEM

8.1. Ellenőrző paraméterek

Anyagnyilatkozat nem szükséges.

8.2. Kitettség ellenőrzés

Szem/arc védelem: kémiai védőszemüveg használata ajánlatos. Tisztítsuk le a szennyezett védőszemüveget újrahasználat előtt.

Kézvédelem: kémiailag ellenálló védőkesztyű használata ajánlatos. Tisztítsuk le a szennyezett védőkesztyűt újrahasználat előtt.

Egyéb személyes védőruha: intézkedéseket kell tenni az anyagok szembe vagy bőrre fröccsenésének megakadályozására.

Viseljünk szemvédőt és védőruházatot.

Légzésvédelem: nem megfelelő szellőzés esetén alkalmazzunk megfelelő légzőkészüléket.

Rövid ideig tartó kitettség esetén használjunk szűrőberendezést

Bőrvédelem: könnyű védőruha ajánlatos. Tisztítsuk le a szennyezett ruházatot újrahasználat előtt.

Kerüljük el a belélegzést és a bőrrel és szemmel való érintkezést.

Technikai ellenőrzés-szellőzés: ne lélegezzünk be gőzöket.

Mechanikai elszívás szükséges: korlátozottan vagy gyengén szellőztetett területeken megfelelő légzőkészülék használatára szükség lehet.

9. FIZIKAI ÉS KÉMIAI TULAJDONSÁGOK

9.1. Információ az alapvető fizikai és kémiai tulajdonságokról

Külső jellemzők

Forma:	folyékony
Szín:	narancssárga
Szag:	jellegzetes
Szagküszöbérték:	Nincs meghatározva
Ph érték:	Nincs meghatározva
Olvadáspont/olvadási tartomány:	Nem meghatározható
Lobbanáspont:	80℃
Gyúlékonyság (szilárd, gázhalmazállapotú):	Nem alkalmazható
Gyulladási hőmérséklet:	Nincs meghatározva
Bomlási hőmérséklet:	Nincs meghatározva
Öngyulladás:	Nincs meghatározva
Robbanásveszély:	Nincs meghatározva
Robbanási határok:	
	Alsó: Nincs meghatározva
	Felső: Nincs meghatározva

Aromax Zrt.

Sűrűség 20℃-nál:	1,026 g/cm ^{3}
Relatív sűrűség	Nincs meghatározva
Gőzsűrűség	Nincs meghatározva
Párolgási sebesség	Nincs meghatározva
Oldhatóság/keverhetőség az alábbiakkal	
	Víz: Egyáltalán nem, vagy csak kismértékben keverhető
Eloszlási együttható (n-Oktanol/víz)	Nincs meghatározva
Egyéb információ	További információk nem állnak rendelkezésre

10. STABILITÁS ÉS REAKCIÓKÉSZSÉG

Reakciókészség

Kémiai stabilitás

Termikus bomlás/kerülendő feltételek: Rendeltetésszerű használat esetén nincs bomlás.

A veszélyes reakciók lehetősége. Veszélyes reakciók nem ismeretesek

Kerülendő körülmények: További lényeges információk nem állnak rendelkezésre

Nem összeférhető anyagok: További lényeges információk nem állnak rendelkezésre

Veszélyes bomlástermékek: Lényeges információk nem állnak rendelkezésre

11. TOXIKOLÓGIAI INFORMÁCIÓ

11.1 Információ a toxikológiai hatásokról:

Akut toxicitás:

Primer ingerhatás:

A bőrön: Ingerli a bőrt és a nyálkahártyát

A szemben: Nem lép fel ingerlő hatás

Érzékenyítés: Bőrrel való érintkezés esetén a szenzibilizáció lehetséges

További toxikológiai információk:

Készítményekre vonatkozó általános besorolási irányelvek érvényes kiadásban közölt számítási eljárás alapján. A termék az alábbi veszélyekkel jár:

A vegyszerekkel kapcsolatos rendelkezés B Mellékletének érvényes kiadásában közölt számítási eljárás szerint a termék az alábbi veszélyekkel jár:

Irritatív, nagyon mérgező,mérgező,ártalmas, maró

A bőrbe történő beszívódás veszélyt jelent. Helyi jellegű ingerlések mellett nagy koncentráció belélegzése esetén mindenek előtt narkotikus hatás lép fel a központi légzőrendszer bénulásának különös veszélyével.

Aromax Zrt.

Helyi jellegű ingerlésjelenségek mellett elsősorban narkotikus hatás jön létre nagyobb koncentrációk belélegzése esetén é a központi légzőrendszer bénulásának veszélye is fenn áll.

Az anyag lenyelése erős maróhatást fejt ki a szájban és a gégében, valamint a nyelőcső és a gyomor perforációjának veszélyével jár.

12. ÖKOLÓGIAI INFORMÁCIÓK

Toxicitás

Akvatikus toxicitás. További lényeges információ nem áll a rendelkezésünkre

Bomlékonyság

További lényeges információ nem áll a rendelkezésünkre

Bioakkumulációs potenciál

További lényeges információ nem áll a rendelkezésünkre

Terjedés a talajban

További lényeges információ nem áll a rendelkezésünkre

További ökológiai információk:

Általános információk:

2(saját besorolás)

Vízveszélyeztetési osztály: vizeket veszélyezteti

Ne engedjük bele a talajvízbe, a környezeti vizekbe, vagy a csatornahálózatba.

Csekély mennyiségek talajba kerülése is veszélyezteti az ivóvizet

Vízi élőlényekre káros

PBT és vPvB értékelési eredmények

Nem alkalmazható

13. HULLADÉKKEZELÉS, ÁRTALMATLANÍTÁS

Kezelési feltételek

Nem keverhető hozzá a háztartási hulladékhoz. Ne engedjük bele a csatornahálózatba

Tisztítatlan csomagolások

A kezelés módját a hatósági előírások szabják meg

14. SZÁLLÍTÁSRA VONATKOZÓ ELŐÍRÁSOK

Aromax Zrt.

UN szám

ADR,ADN,IMDG,IATA Érvénytelen

Megfelelő szállítási név:

ADR,ADN,IMDG,IATA Érvénytelen

Szállítási veszélyességi osztály(ok)

ADR,ADN,IMDG,IATA

Osztály Érvénytelen

Csomagolási csoport:

ADR,IMDG, IATA Érvénytelen

Környezeti veszélyek:

Marina pollutant: Nem

A felhasználót érintő különleges óvintézkedések: Nem alkalmazható

A MARPOL 73/78 II.melléklete és az IBC kódex szerinti ömlesztett szállítás

Nem alkalmazható

UN"Model Regulation" Nem alkalmazható

15. SZABÁLYOZÁSI INFORMÁCIÓK

Az adott anyaggal vagy keverékkel kapcsolatos biztonsági, egészségügyi és környezetvédelmi előírások/jogszabályok

Az 1272/2008/EK rendelet szerinti cimkézés

A termék a CLP-rendelet (Anyagok és Keverékek Osztályozásáról, Cimkézéséről és Csomagolásáról szóló rendelet) szerint nincs osztályozva és címkézve

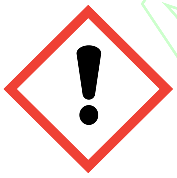

Figyelmeztető veszély

Veszélyt megahtározó komponensek a cimkézéshez

Fahéjaldehid

Eugenol

Trans-Anethole

Aromax ZRt.

DIPENTENE

Veszélyességi intézkedés(ek):

H317 Allergiás bőrreakciót válthat ki
H312 Bőrrel érintkezve ártalmas
H319 Súlyos szemirritációt válthat ki
H315 Bőrirritáló hatású
H412 Ártalmas a vízi élővilágra, hosszan tartó károsodást okoz

Elővigyázatossági intézkedések:

P261 Kerülje a por/füst/gáz/köd/gőzök/permet belélegzését
P280 Védőkesztyű/védőruha/szemvédő/arcvédő használata kötelező
P305+P351+P338 Szembe kerülés esetén: Több percig tartó óvatos öblítés vízzel. Adott esetben a kontaklencsék eltávolítása, ha könnyen megoldható. Az öblítés folytatása
P321 Szakellátás (lásd a címkén)
P322 Különleges intézkedések (lásd a címkén)
P501 Rendelkezzen a tartalomról/tárolásról a helyi/regionális/nemzeti/nemzetközi előírásoknak megfelelően

Egyéb veszélyek:
A PBT és vPvB értékelés eredményei
PBT: Nem alkalmazható
vPvB: Nem alkalmazható

16. EGYÉB INFORMÁCIÓK

Kockázatok
H226 Tűzveszélyes folyadék és gőz.
H302 Lenyelve ártalmas
H312 Bőrrel érintkezve ártalmas
H304 Halálos lehet lenyelve és a légutakba kerülve.
H315 Bőrirritáló hatású.
H316 A bőrre enyhén irritáló hatású
H317 Allergiás bőrreakciót válthat ki.

AromaxZRt.

H319 Súlyos szemirritációt okoz

H400 Nagyon mérgező a vízi élővilágra

H410 Nagyon mérgező a vízi élővilágra, hosszan tartó károsodást okoz

H411 Mérgező a vízi élővilágra, hosszan tartó károsodást okoz

R10 Kis mértékben tűzveszélyes

R21 Bőrrel érintkezve ártalmas

R22 Lenyelve ártalmas

R36 Szemizgató hatású

R38 Bőrizgató hatású

R43 Bőrrel érintkezve túlérzékenységet okozhat (szenzibilizáló hatású lehet)

R50/53 Nagyon mérgező a vízi szervezetekre, a vízi környezetben hosszan tartó károsodást okozhat

R51/53 Mérgező a vízi szervezetekre, a vízi környezetben hosszan tartó károsodást okozhat

R65 Lenyelve ártalmas, aspiráció (Idegen anyagnak a légutakba beszívása) esetén tüdőkárosodást okozhat

A megadott információk jelen tudásunkon alapulnak és a termék szállított állapotára vonatkoznak. Az adatlap csak a biztonsági követelmények szempontjából jellemzi a terméket, és nem arra szolgál, hogy annak bizonyos tulajdonságait garantálja. Nem helyettesíti a termékspecifikációt. Az érvényes rendeletek betartása, az esetleges speciális felhasználású technológiának megfelelő biztonsági előírások kialakítása és betartása a felhasználó feladata. Az anyaggal (készítménnyel) kapcsolatos konkrét felhasználási mód során a 98/24/EK irányelv alapján fel kell mérni és értékelni kell a munkavállalók egészségét és biztonságát veszélyeztető kockázatokat.

Aromax ZRt.

Kiadás száma: 1.2
Kiadás kelte: 2012.11.22.
Felülvizsgálat kelte: 2013.03.21.

1. AZONOSÍTÁS

Anyag/készítmény megnevezése:

- magyar név: Szegfűszeg olaj
- angol név: Clove oil
- INCI:

Felhasználási területek: Illatszerek, kozmetika
CAS-szám: 84961-50-2
EINECS: 284-638-7
Gyártó/forgalmazó adatai:

- Cégnév: Aromax Zrt.
- Cím: 1031 Budapest Záhony utca 7
- Tel: +36 1 8808 480 Fax: +36 1 8808 481 E-mail: aromax@aromax.hu

Egészségügyi Toxikológiai Tájékoztató Szolgálat: 1096 Budapest, Nagyvárad tér 2.
Tel: +36 80 201 199 Fax: +36 1 476-1138

2. VESZÉLYEK AZONOSÍTÁSA

Az anyag vagy keverék osztályozása:

Az 1272/2008/EK rendelet szerinti osztályozás

Eye Irrit. 2 H319 Súlyos szemirritációt okoz
Skin Sens. 1 H317 Allergiás bőrreakciót válthat ki.

A 67/548/EGK irányelv vagya 1999/45/EK irányelv szerinti osztályozás
Xi. Irritatív
R36: Szemizgató hatású
Xi; Túlérzékenységet okozó
R43 Bőrrel érintkezve túlérzékenységet okozhat (szenzibiláló hatású lehet)
Az emberek és a környezet veszélyeztetettségére vonatkozó különleges információk
A termék jelölése a többször módosított „Készítmények általános besorolási EK-irányelvei” számítási eljárása értelmében kötelező
Osztályozási rendszer:
Az osztálybasorolás megfelel az érvényes EK listáknak, de kiegészítik a szakirodalomból származó és a cégek által megadott adatok.

Címkézési elemek:

Aromax Zrt.

Az 1272/2008/EK rendelet szerinti cimkézés
A termék a CLP-rendelet (Anyagok és Keverékek Osztályozásáról, Cimkézéséről és Csomagolásáról szóló rendelet) szerint nincs osztályozva és címkézve

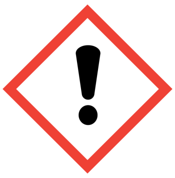

Figyelmeztetés: Figyelem
Veszélyt megahtározó komponensek a cimkézéshez
Eugenol

Veszélyességi intézkedés(ek):

H317 Allergiás bőrreakciót válthat ki
H319 Súlyos szemirritációt koz

Elővigyázatossági intézkedések:

P261 Kerülje a por/füst/köd/gőzök/permet belélegzését
P280 Védőkesztyű/védőruha/szemvédő/arcvédő használata kötelező
P305+P351+P338 SZEMBE KERÜLÉS esetén: Több percig tartó óvatos öblítés vízzel. Adott esetben a kontaktlencsék eltávolítása, ha könnyen megoldható. Az öblítés folytatása
P321 Szakellátás (lásd a címkén)
P363 A szennyezett ruhát újboli használat előtt ki kell mosni
P501 Rendelkezzen a tartalomról/tárolásról a helyi/regionális/nemzeti/nemzetközi előírásoknak megfelelően

Egyéb veszélyek:
A PBT és vPvB értékelés eredményei
PBT: Nem alkalmazható
vPvB: Nem alkalmazható

3. ÖSSZETÉTELRE VONATKOZÓ INFORMÁCIÓK

Kémiai jellemzés:Keverékek
CAS: 84961-50-2
EINECS: 284-638-7
Leírás: Az alábbi anyagokból álló, veszélytelen adalékokat tartalmazó keverék

Aromax ZRt.

Veszélyes alkotórészek:

**Kémiai név**	**Tartalom (%)**	**CAS szám**	**Veszélyesség***	**Osztályozás (Direktíva 67/548/EEC)***
Eugenol	50-100	97-53-0	Eye Irrit. 2, H319;Skin Sens. 1, H317	Xi R36; Xi R43
Abeta-Caryophyllene	5-9,99	87-44-5	Asp. Tox. 1, H304	Xn R65

*A termékre vonatkozó veszélyességi utalások szövege a 16. fejezetben található.

4. ELSŐSEGÉLYNYÚJTÁSI INTÉZKEDÉSEK

4.1. A szükséges elsősegélynyújtási intézkedések

Belégzés esetén:	A sérültet friss levegőre kell vinni. Ha nem lélegzik, mesterséges lélegeztetést kell alkalmazni. Nehézlégzés esetén adjunk oxigént. Azonnal hívjunk orvost.
Bőrrel való érintkezés esetén:	A szennyezett ruházatot távolítsuk el, a szennyeződött bőrfelületet bő vízzel és szappannal mossuk le. Ha a tünetek fennmaradnak, hívjunk orvost.
Szembe kerülés esetén:	Öblítsük bő langyosvízzel legalább 15 percen keresztül, a szemhéjakat szélesre nyitva. Hívjunk szemorvost.
Lenyelés esetén:	Öblítsük ki a szánkat hidegvízzel, és forduljunk orvoshoz. Maradjunk nyugalmi helyzetben, ne próbáljunk hányni.

4.2. A legfontosabb akut és krónikus tünetek és hatások

Nem áll rendelkezésre információ.

4.3. Bármely azonnal szükséges orvosi odafigyelés és speciális kezelés

Nem áll rendelkezésre információ.

Aromax Zrt.

5. TŰZVÉDELMI INTÉZKEDÉSEK

5.1. Oltóközeg

Víz spray, széndioxid, száraz kémiai por vagy megfelelő hab

Biztonságtechnikai okokból ne használjunk teljes vízsugarat.

5.2. Vegyület vagy keverék használatából adódó speciális kockázatok

Ismert módon vagy várhatóan veszélyes éghető termékek: égés során toxikus füstöt bocsátanak ki.

5.3. Tűzoltási tanácsok

Egyéb tűzoltási megfontolások: a magas hőmérséklet zárt tartályokon belüli magas nyomáshoz vezethet.

Kerüljük a keletkező gőzök belélegzését. Használjunk légzőkészüléket. A tűz ne terjedhessen szennyvízcsatornákba és vízfolyásokba.

Speciális tűzvédelmi eljárások: viseljünk önhordó légzőkészüléket és védőruhát, hogy megelőzhessük a bőrrel és szemmel való érintkezést.

.

6. ÓVINTÉZKEDÉSEK BALESET (VÉLETLEN KIÖMLÉS) ESETÉRE

6.1. Személyes elővigyázatosság, védőberendezések és sürgősségi eljárások

Nem szükséges

6.2. Környezetvédelmi óvintézkedések

Hogy megelőzzük a környezet esetleges elszennyeződését, ne hagyjuk, hogy az anyag szennyvízcsatornába, felszíni vizekbe vagy a talajvízbe jusson.

6.3. A területi elhatárolás és szennyezésmentesítés módszerei és anyagai:

Folyadékot megkötő anyaggal (homok, kovaföld, savmegkötő anyag, univerzális megkötő anyag) itassuk fel.

A szennyezett anyagot, mint hulladékot a 13. pont szerint távolítsuk el.

Kezeljük 2%-os nátronlóggal

Gondoskodjunk megfelelő szellőztetésről

6.4. Más pontokra hivatkozás

A biztonságos kezeléshez lásd a 7. fejezetben közölt információkat.

A személyes védőfelszereléshez lásd a 8. fejezetben közölt információkat

Az eltávolítással kapcsolatban lásd a 13. fejezetben közölt információkat

7. KEZELÉS ÉS TÁROLÁS

Kezelés

A biztonságos kezelésre irányuló óvintézkedések. Kerüljük az aerosol képződést.

Zűz- és robbanásvédelmi információk: Különleges intézkedés nem szükséges.

A biztonságos tárolás feltételei, az esetleges összeférhetetlenséggel együtt

Raktározás:

A raktárhelyiségekkel és tartályokkal szemben támasztott követelmények: Nincsenek különleges követelmények.

Együttes tárolással kapcsolatos információk: Nem szükséges

Aromax Zrt.

További adatok a raktározási körülményekkel kapcsolatban: Az edényeket jól lezárt állapotban tartsuk.

Meghatározott végfelhasználás (végfelhasználások): További lényeges információk nem állnak rendelkezésre.

8. EXPOZÍCIÓ ELLENŐRZÉSE / SZEMÉLYI VÉDELEM

Pótlólagos információ a műszaki berendezés kialakításához: További adatok nincsenek. Lásd. 7. pont

8.1. Ellenőrző paraméterek

Alkotórészek munkahelyre vonatkoztatott, felügyelet tárgyát képező határértékkel:

A termék nem tartalmaz olyan releváns anyagmennyiségeket, amelyek munkahelyre vonatkoztatott, ellenőrizendő határértékekkel rendelkeznek

Pótlólagos információk: A létrehozásnál érvényes listák képezték a kiindulópontot.

Az expozíció ellenőrzése

Személyes védőfelszerelés:

Általános védekezési és higiéniai intézkedések:

Tartsuk távol élelmiszerektől, italoktól és takarmánytól.

A szennyezett folyadékkal átitatott ruházatot azonnal vegyük le

Munkahelyi szünetek előrr és a munka befejezésekor mossunk kezet.

Kerüljük a szemmel és bőrrel való érintkezést.

Légzésvédelem: Nem megfelelő szellőzéskor védőálarc szükséges.

Kézvédelem:

Védőkesztyű.

A kesztyű anyagának át nem eresztő képességűnek és a termékkel/anyaggal/készítménnyel szemben ellenállónak kell lennie.

Kesztyűanyag:

A vízben oldhatatlan anyag/termék/készítmény használata előtt vízoldékony bőrvédő szert (zsírmentes rétegképzőt vagy olaj/víz-emulziót) használjunk. Mivel a termék több anyagból összeállított készítményt jelent, a kesztyő anyagánka tartóssága előre nem számítható ki, ezért a használat előtt ezt ellenőrizni kell.

Barrier 02- 100

Áthatolási idő a kesztyűanyagon. A pontos behatolási időt a kesztyű gyártójától kell megkérdezni és azt be kell tartani.

Szemvédelem:

Védőszemüveg:

Jól záródó védőszemüveg

Aromax ZRt.

9. FIZIKAI ÉS KÉMIAI TULAJDONSÁGOK

Információ az alapvető fizikai és kémiai tulajdonságokról

Külső jellemzők

Forma: folyékony

Szín: sárgás

Szag: jellegzetes

Szagküszöbérték: Nincs meghatározva

Ph érték: Nincs meghatározva

Olvadáspont/olvadási tartomány: Nem meghatározható

Lobbanáspont: 110℃

Gyúlékonyság (szilárd, gázhalmazállapotú): Nem alkalmazható

Gyulladási hőmérséklet: Nincs meghatározva

Bomlási hőmérséklet: Nincs meghatározva

Öngyulladás: Nincs meghatározva

Robbanásveszély: Nincs meghatározva

Robbanási határok:

Alsó: Nincs meghatározva

Felső: Nincs meghatározva

Sűrűség 20℃-nál: 1,043 g/cm^3

Relatív sűrűség Nincs meghatározva

Gőzsűrűség Nincs meghatározva

Párolgási sebesség Nincs meghatározva

Oldhatóság/keverhetőség az alábbiakkal

Víz: Egyáltalán nem, vagy csak kismértékben keverhető

Eloszlási együttható (n-Oktanol/víz) Nincs meghatározva

Egyéb információ További információk nem állnak rendelkezésre

10. STABILITÁS ÉS REAKCIÓKÉSZSÉG

Reakciókészség

Kémiai stabilitás

Termikus bomlás/kerülendő feltételek: Rendeltetésszerű használat esetén nincs bomlás.

A veszélyes reakciók lehetősége. Veszélyes reakciók nem ismeretesek

Kerülendő körülmények: További lényeges információk nem állnak rendelkezésre

Nem összeférhető anyagok: További lényeges információk nem állnak rendelkezésre

Aromax Zrt.

Veszélyes bomlástermékek: Lényeges információk nem állnak rendelkezésre

11. TOXIKOLÓGIAI INFORMÁCIÓ

Információ a toxikológiai hatásokról:

Akut toxicitás:

Primer ingerhatás:

A bőrön: Nem fejt ki ingerlő hatást

A szemben: Ingerlő hatás

Érzékenyítés: Bőrrel való érintkezés esetén a szenzibilizáció lehetséges

További toxikológiai információk:

Készítményekre vonatkozó általános besorolási irányelvek érvényes kiadásban közölt számítási eljárás alapján. A termék az alábbi veszélyekkel jár:

A vegyszerekkel kapcsolatos rendelkezés B Mellékletének érvényes kiadásában közölt számítási eljárás szerint a termék az alábbi veszélyekkel jár:

Irritatív

12. ÖKOLÓGIAI INFORMÁCIÓK

Toxicitás

Akvatikus toxicitás: Nem áll rendelkezésre több lényeges információ

Perzisztencia és lebonthatóság: Nem áll rendelkezésre több lényeges információ

Környezeti feltételek melleti viselkedés:

Bioakkumulációs képesség: Nem áll rendelkezésre több lényeges információ

A talajban való mobilitás: Nem áll rendelkezésre több lényeges információ

Ökotoxikus hatások:

Megjegyzés: A vizsgált koncentrációig halakra nem ártalmas

További ökológiai információk:

Általános információk:

2 (saját besorolás) Vízveszélyetzetési osztály: a vizeket veszélyezteti.

Ne engedjük bele a talajvízbe, a környezeti vizekbe, vagy a csatornahálózatba. Csekély mennyiségek talajba kerülése is veszélyezteti az ivóvizet.

A PBT- és a vPvB-értékelés eredményei

PBT: Nem alkalmazható

vPvB: Nem alkalmazható

Egyéb káros hatások: Nem áll rendelkezésre több lényeges információ

Aromax Zrt.

13. HULLADÉKKEZELÉS, ÁRTALMATLANÍTÁS

Hulladékkezelési módszerek

Ajánlás: Nem keverhető hozzá a háztartási hulladékhoz. Ne engedjük bele a csatornahálzatba

Tisztítatlan csomagolások:

Ajánlás: A kezelés módját a hatósági előírások szabják meg

14. SZÁLLÍTÁSRA VONATKOZÓ ELŐÍRÁSOK

**UN szám**	
ADR,ADN,IMDG,IATA	Érvénytelen
**Az ENSZ szerint megfelelő szállítási megnevezés**	
ADR,ADN,IMDG,IATA	Érvénytelen
**Szállítási veszélyességi osztály(ok)**	
ADR,ADN,IMDG,IATA	
Osztály	Érvénytelen
**Csomagolási csoport**	
ADR, IMDG,IATA	Érvénytelen
**Környezeti veszélyek:**	
Marine pollutant	Nem
**A felhasználót érintő különleges óvintézkedések**	Nem alkalmazható
**A MARPOL 73/78 II. melléklete és az IBC kódex szerinti ömlesztett szállítás**	Nem alkalmazható
**UN „Model Regulation”**	-

15. SZABÁLYOZÁSI INFORMÁCIÓK

Az adott anyaggal vagy keverékkel kapcsolatos biztonsági, egészségügyi és környezetvédelmi előírások/jogszabályok

Az 1272/2008/EK rendelet szerinti cimkézés

A termék a CLP-rendelet (Anyagok és Keverékek Osztályozásáról, Cimkézéséről és Csomagolásáról szóló rendelet) szerint nincs osztályozva és címkézve

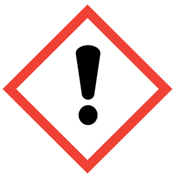

Figyelmeztető veszély

Aromax Zrt.

Veszélyt meghatározó komponensek a cimkézéshez
Eugenol

Veszélyességi intézkedés(ek):

H319 Súlyos szemirritációt okoz
H317 Allergiás bőrreakciót válthat ki

Elővigyázatossági intézkedések:

P261 Kerülje a por/füst/köd/gőzök/permet belélegzését
P280 Védőkesztyű/védőruha/szemvédő/arcvédő használata kötelező
P305+P351+P338 SZEMBE KERÜLÉS esetén: Több percig tartó óvatos öblítés vízzel. Adott esetben a kontaklencsék eltávolítása, ha könnyen megoldható. Az öblítés folytatása
P321 Szakellátás (lásd a címkén)
P363 A szennyezett ruhát újboli használat előtt ki kell mosni
P501 Rendelkezzen a tartalomról/tárolásról a helyi/regionális/nemzeti/nemzetközi előírásoknak megfelelően

16. EGYÉB INFORMÁCIÓK

Az adatok jelenlegi ismereteinkre támaszkodnak, azonban nem jelentik a termék tulajdonságainak garanciáját és nem alapoznak meg szerződéses jogviszonyt.

Lényeges mondatok
H304 Halálos lehet lenyelve és a légutakba kerülve.
H317 Allergiás bőrreakciót válthat ki.
H319 Súlyos szemirritációt okoz

R36 Szemizgató hatású
R43 Bőrrel érintkezve túlérzékenységet okozhat (szenzibilizáló hatású lehet)
R65 Lenyelve ártalmas, aspiráció (Idegen anyagnak a légutakba beszívása) esetén tüdőkárosodást okozhat

A megadott információk jelen tudásunkon alapulnak és a termék szállított állapotára vonatkoznak. Az adatlap csak a biztonsági követelmények szempontjából jellemzi a terméket, és nem arra szolgál, hogy annak bizonyos tulajdonságait garantálja. Nem helyettesíti a termékspecifikációt. Az érvényes rendeletek betartása, az esetleges speciális felhasználású technológiának megfelelő biztonsági előírások kialakítása és

Aromax Zrt.

betartása a felhasználó feladata. Az anyaggal (készítménnyel) kapcsolatos konkrét felhasználási mód során a 98/24/EK irányelv alapján fel kell mérni és értékelni kell a munkavállalók egészségét és biztonságát veszélyeztető kockázatokat.

Aromax ZRt.
