# Supplementary material for: Development of a novel, entirely herbal-based mouthwash effective against common oral bacteria and SARS-CoV-2
Source: BMC Complement Med Ther. 2023 May 1;23:138. doi: 10.1186/s12906-023-03956-3 (PMC10150350; doi:10.1186/s12906-023-03956-3)
Supplement: Supplementary file 4 — Additional file 4. Summary table and references for the active oily compounds and their presence in the selectedplants and related species. A summary table, references and PubChem CID of the active oily compounds and their presence in the selected plants and related species. [file 12906_2023_3956_MOESM4_ESM.docx]

| **Compound** | **PubChem CID** | **Taxonomic presence in the selected plants and related species** | **References** |
| --- | --- | --- | --- |
| cinnamaldehyde | 637511 | *Cinnamomum sp.* | [1-7] |
| cinnamyl acetate | 5282110 | *Cinnamomum sp.* | [1, 8, 9] |
| eugenol | 3314 | *Hibiscus sp.* | [10] |
|  |  | *Lavandula sp.* | [11] |
|  |  | *Cinnamomum sp.* | [1, 6, 8, 12-15] |
|  |  | *Quercus sp.* | [16] |
|  |  | *Pimpinella sp.* | [17] |
|  |  | *Mentha sp.* | [18, 19] |
| linalool | 6549 | *Hypericum sp.* | [20] |
|  |  | *Eugenia sp.* | [21] |
|  |  | *Cinnamomum sp.* | [8, 22] |
|  |  | *Lavandula sp.* | [11, 23-25] |
|  |  | *Quercus sp.* | [16, 26] |
|  |  | *Juglans sp.* | [27] |
|  |  | *Mentha sp.* | [28-30] |
|  |  | *Pimpinella sp.* | [31] |
| beta-caryophyllene | 5281515 | *Eugenia sp.* | [21] |
|  |  | *Cinnamomum sp.* | [1, 15] |
|  |  | *Pimpinella sp.* | [31] |
|  |  | *Mentha sp.* | [19, 29, 32-34] |
|  |  | *Lavandula sp.* | [11, 23, 24, 35] |
|  |  | *Juglans sp.* | [27] |
|  |  | *Hypericum sp.* | [36, 37] |
| 1,8-cineole | 2758 | *Cinnamomum sp.* | [13] |
|  |  | *Mentha sp.* | [28, 38, 39] |
|  |  | *Lavandula sp.* | [40, 41] |
| trans-anethole | 637563 | *Cinnamomum sp.* | [14] |
|  |  | *Pimpinella sp.* | [17, 42-46] |
| alpha-pinene | 6654 | *Cinnamomum sp.* | [8, 13, 22] |
|  |  | *Quercus sp.* | [26] |
|  |  | *Pimpinella sp.* | [17, 31] |
|  |  | *Mentha sp.* | [19, 28, 33, 47, 48] |
|  |  | *Lavandula sp.* | [23, 49, 50] |
|  |  | *Juglans sp.* | [27, 51, 52] |
|  |  | *Hypericum sp.* | [53-56] |
| beta-pinene | 14896 | *Cinnamomum sp.* | [22, 57] |
|  |  | *Quercus sp.* | [26] |
|  |  | *Pimpinella sp.* | [31] |
|  |  | *Mentha sp.* | [28] |
|  |  | *Lavandula sp.* | [23, 40, 58-60] |
|  |  | *Juglans sp.* | [27, 52, 61] |
|  |  | *Hypericum sp.* | [36, 37, 53-56] |
| carvacrol | 10364 | *Cinnamomum sp.* | [8, 13, 14] |
|  |  | *Pimpinella sp.* | [62] |
|  |  | *Mentha sp.* | [19, 63] |
|  |  | *Lavandula sp.* | [58, 64] |
|  |  | *Juglans sp.* | [51] |
| para-cymene | 7463 | *Cinnamomum sp.* | [8, 22] |
|  |  | *Quercus sp.* | [26] |
|  |  | *Pimpinella sp.* | [31, 62] |
|  |  | *Mentha sp.* | [28, 39] |
|  |  | *Lavandula sp.* | [11, 23, 49, 59, 65-67] |
|  |  | *Hypericum sp.* | [53] |
| para-mentha-1,4-dien | 7461 | *Eugenia sp.* | [68] |
|  |  | *Mentha sp.* | [32, 33, 63, 69, 70] |
|  |  | *Pimpinella sp.* | [31, 62] |
|  |  | *Cinnamomum sp.* | [1, 22] |
|  |  | *Lavandula sp.* | [23, 49, 66] |
|  |  | *Hypericum sp.* | [53] |
|  |  | *Juglans sp.* | [61] |
|  |  | *Quercus sp.* | [26] |

Additional File 2. Summary table and references for the active oily compounds and their presence in the selected plants and related species.

**References for this table:**

[1] S. Fujita, [Miscellaneous contributions to the essential oils of plants from various territories. XLVII. On the components of essential oils of Cinnamomum sieboldii Meisn], Yakugaku Zasshi 106(1) (1986) 17-21.

[2] S.S. Lin, T.M. Lu, P.C. Chao, Y.Y. Lai, H.T. Tsai, C.S. Chen, Y.P. Lee, S.C. Chen, M.C. Chou, C.C. Yang, In vivo cytokine modulatory effects of cinnamaldehyde, the major constituent of leaf essential oil from Cinnamomum osmophloeum Kaneh, Phytother Res 25(10) (2011) 1511-8.

[3] A.M. Reddy, J.H. Seo, S.Y. Ryu, Y.S. Kim, K.R. Min, Y. Kim, Cinnamaldehyde and 2-methoxycinnamaldehyde as NF-kappaB inhibitors from Cinnamomum cassia, Planta Med 70(9) (2004) 823-7.

[4] H.S. Lee, Y.J. Ahn, Growth-Inhibiting Effects of Cinnamomum cassia Bark-Derived Materials on Human Intestinal Bacteria, J Agric Food Chem 46(1) (1998) 8-12.

[5] Y.Y. Ma, H.R. Huo, C.H. Li, B.S. Zhao, L.F. Li, F. Sui, S.Y. Guo, T.L. Jiang, Effects of cinnamaldehyde on PGE2 release and TRPV4 expression in mouse cerebral microvascular endothelial cells induced by interleukin-1beta, Biol Pharm Bull 31(3) (2008) 426-30.

[6] H.S. Lee, B.S. Kim, M.K. Kim, Suppression effect of Cinnamomum cassia bark-derived component on nitric oxide synthase, J Agric Food Chem 50(26) (2002) 7700-3.

[7] S.Y. Tsai, S.C. Chen, A fluorometric assay of trans-cinnamaldehyde in cinnamon, J Nat Prod 47(3) (1984) 536-8.

[8] J.E. Angmor, D.M. Dicks, W.C. Evans, D.K. Santra, Studies on Cinnamomum zeylanicum, Planta Med 21(4) (1972) 416-20.

[9] G.K. Jayaprakasha, L.J. Rao, K.K. Sakariah, Chemical composition of volatile oil from Cinnamomum zeylanicum buds, Z Naturforsch C J Biosci 57(11-12) (2002) 990-3.

[10] B.H. Ali, N. Al Wabel, G. Blunden, Phytochemical, pharmacological and toxicological aspects of Hibiscus sabdariffa L.: a review, Phytother Res 19(5) (2005) 369-75.

[11] G. Gabbrielli, F. Loggini, P.L. Cioni, B. Giannaccini, E. Mancuso, Activity of lavandino essential oil against non-tubercular opportunistic rapid grown mycobacteria, Pharmacol Res Commun 20 Suppl 5 (1988) 37-40.

[12] S. Prabuseenivasan, M. Jayakumar, S. Ignacimuthu, In vitro antibacterial activity of some plant essential oils, BMC Complement Altern Med 6 (2006) 39.

[13] P.M. Giang, W.A. König, P.T. Son, Chemical constituents of the essential oil from the bark of Cinnamomum illicioides A. Chev. from Vietnam, J Nat Med 60(3) (2006) 248-250.

[14] B. Shan, Y.Z. Cai, M. Sun, H. Corke, Antioxidant capacity of 26 spice extracts and characterization of their phenolic constituents, J Agric Food Chem 53(20) (2005) 7749-59.

[15] S.Y. Kuo, T.J. Hsieh, Y.D. Wang, W.L. Lo, Y.R. Hsui, C.Y. Chen, Cytotoxic constituents from the leaves of Cinnamomum subavenium, Chem Pharm Bull (Tokyo) 56(1) (2008) 97-101.

[16] H.A. Palma-Fleming, R.E. Kepner, Volatile components of california live oak, quercus agrifolia, Phytochemistry 22(6) (1983) 1503-1505.

[17] N.P. Mekhtieva, Essential oils of Pimpinella aromatica, Chemistry of Natural Compounds 27(2) (1991) 249-251.

[18] I.J.M. Merks, A.B. Svendsen, Occurrence and Possible Role of Glycosidic Bound Eugenol and 2-Methoxy-4-Vinylphenol in the Lignin Biosynthesis of Some Lamiaceae.

[19] M.D.P. Raya, M.P. Utrilla, M.C. Navarro, J. Jiménez, CNS activity of Mentha rotundifolia and Mentha longifolia essential oil in mice and rats, Phytotherapy Research 4(6) (1990) 232-234.

[20] I. Schwob, J.M. Bessiere, M. Dherbomez, J. Viano, Composition and antimicrobial activity of the essential oil of Hypericum coris, Fitoterapia 73(6) (2002) 511-3.

[21] T.R. Costa, O.F. Fernandes, S.C. Santos, C.M. Oliveira, L.M. Lião, P.H. Ferri, J.R. Paula, H.D. Ferreira, B.H. Sales, R. Silva M do, Antifungal activity of volatile constituents of Eugenia dysenterica leaf oil, J Ethnopharmacol 72(1-2) (2000) 111-7.

[22] U.M. Senanayake, T.H. Lee, R.B.H. Wills, Volatile constituents of cinnamon (Cinnamomum zeylanicum) oils, Journal of Agricultural and Food Chemistry 26(4) (1978) 822-824.

[23] P.R. Venskutonis, A. Dapkevicius, M. Baranauskiene, Composition of the Essential Oil of Lavender (Lavandula angustifolia Mill.) from Lithuania, Journal of Essential Oil Research 9(1) (1997) 107-110.

[24] G. Buchbauer, L. Jirovetz, W. Jäger, H. Dietrich, C. Plank, Aromatherapy: evidence for sedative effects of the essential oil of lavender after inhalation, Z Naturforsch C J Biosci 46(11-12) (1991) 1067-72.

[25] D. Shaw, J.M. Annett, B. Doherty, J.C. Leslie, Anxiolytic effects of lavender oil inhalation on open-field behaviour in rats, Phytomedicine 14(9) (2007) 613-20.

[26] F. Loreto, P. Ciccioli, E. Brancaleoni, A. Cecinato, M. Frattoni, T.D. Sharkey, Different sources of reduced carbon contribute to form three classes of terpenoid emitted by Quercus ilex L. leaves, Proc Natl Acad Sci U S A 93(18) (1996) 9966-9.

[27] R.G. Buttery, R.A. Flath, T.R. Mon, L.C. Ling, Identification of germacrene D in walnut and fig leaf volatiles, Journal of Agricultural and Food Chemistry 34(5) (1986) 820-822.

[28] D. Yadegarinia, L. Gachkar, M.B. Rezaei, M. Taghizadeh, S.A. Astaneh, I. Rasooli, Biochemical activities of Iranian Mentha piperita L. and Myrtus communis L. essential oils, Phytochemistry 67(12) (2006) 1249-55.

[29] N. Mimica-Dukić, B. Bozin, M. Soković, B. Mihajlović, M. Matavulj, Antimicrobial and antioxidant activities of three Mentha species essential oils, Planta Med 69(5) (2003) 413-9.

[30] J.A. Pino, A. Rosado, V. Fuentes, Essential Oil of Mentha citrata Ehrh. Grown in Cuba, Journal of Essential Oil Research 11(4) (1999) 413-414.

[31] R. Ivanić, K. Savin, F.V. Robinson, Essential Oil from Pimpinella serbica Fruits, Planta Med 48(1) (1983) 60-1.

[32] K. Umemoto, Two New Stereoisomers of 1,2-Epoxymenthyl Acetate from self-Pollinated Plant Oils of Mentha rotundifolia, Natural Product Letters 11(3) (1998) 161-165.

[33] A. Nagell, F.W. Hefendehl, [Composition of the essential oil of Mentha rotundifolia (author's transl)], Planta Med 26(1) (1974) 1-8.

[34] D. Nori-Shargh, H. Norouzi-Arasi, S. Mohammadi, M. Mirza, K. Jaimand, Volatile Component of Mentha longifolia (L.) Huds. from Iran, Journal of Essential Oil Research 12(1) (2000) 111-112.

[35] C. Everaerts, Y. Roisin, J.L. Le Quéré, O. Bonnard, J.M. Pasteels, Sesquiterpenes in the frontal gland secretions of nasute soldier termites from New Guinea, J Chem Ecol 19(12) (1993) 2865-79.

[36] M. Couladis, I.B. Chinou, O. Tzakou, P.V. Petrakis, Composition and antimicrobial activity of the essential oil of Hypericum rumeliacum subsp. apollinis (Boiss. & Heldr.), Phytother Res 17(2) (2003) 152-4.

[37] A.P. Guedes, L.R. Amorim, A.M. Vicente, G. Ramos, M. Fernandes-Ferreira, Essential oils from plants and in vitro shoots of Hypericum androsaemum L, J Agric Food Chem 51(5) (2003) 1399-404.

[38] M. Mahboubi, G. Haghi, Antimicrobial activity and chemical composition of Mentha pulegium L. essential oil, J Ethnopharmacol 119(2) (2008) 325-7.

[39] A.K. Singh, V.K. Raina, A.A. Naqvi, N.K. Patra, B. Kumar, P. Ram, S.P.S. Khanuja, Essential oil composition and chemoarrays of menthol mint (Mentha arvensis L. f. piperascens Malinvaud ex. Holmes) cultivars, Flavour and Fragrance Journal 20(3) (2005) 302-305.

[40] D.V. Banthorpe, H.J. Bilyard, D.G. Watson, Pigment formation by callus of Lavandula angustifolia, Phytochemistry 24(11) (1985) 2677-2680.

[41] Y. Cong, P. Abulizi, L. Zhi, X. Wang, Mirensha, Chemical composition of the essential oil of Lavandula angustifolia from Xinjiang, China, Chemistry of Natural Compounds 44 (2009) 810.

[42] I. Kubo, I. Kinst-Hori, Tyrosinase Inhibitors from Anise Oil, Journal of Agricultural and Food Chemistry 46(4) (1998) 1268-1271.

[43] J.W. LeFevre, Isolating trans-Anethole from Anise Seeds and Elucidating Its Structure: A Project Utilizing One- and Two-Dimensional NMR Spectrometry, Journal of Chemical Education 77(3) (2000) 361.

[44] N. Tabanca, S.I. Khan, E. Bedir, S. Annavarapu, K. Willett, I.A. Khan, N. Kirimer, K.H. Baser, Estrogenic activity of isolated compounds and essential oils of Pimpinella species from Turkey, evaluated using a recombinant yeast screen, Planta Med 70(8) (2004) 728-35.

[45] J. Reichling, R. Martin, U. Thron, Production and accumulation of phenylpropanoids in tissue and organ cultures of pimpinella anisum, Zeitschrift für Naturforschung C 43(1-2) (1988) 42-46.

[46] M. Himejima, I. Kubo, Fungicidal activity of polygodial in combination with anethole and indole against Candida albicans, Journal of Agricultural and Food Chemistry 41(10) (1993) 1776-1779.

[47] T. Hirata, S. Murakami, K. Ogihara, T. Suga, Volatile monoterpenoid constituents of the plantlets of Mentha spicata produced by shoot tip culture, Phytochemistry 29(2) (1990) 493-495.

[48] R.H. Eastman, The Isolation of Menthofuran from American Peppermint Oil, Journal of the American Chemical Society 72(11) (1950) 5313-5314.

[49] A.O. Tucker, M.J. Maciarello, S. Angell, J.R. Espaillat, E.C. French, The Essential Oil of Lavandula x hybrida Balb. ex Ging., a Distinct Hybrid from L. x heterophylla Poir. (Labiatae), Journal of Essential Oil Research 5(4) (1993) 443-445.

[50] H. Sebai, S. Selmi, K. Rtibi, A. Souli, N. Gharbi, M. Sakly, Lavender (Lavandula stoechas L.) essential oils attenuate hyperglycemia and protect against oxidative stress in alloxan-induced diabetic rats, Lipids Health Dis 12 (2013) 189.

[51] G. Buchbauer, L. Jirovetz, Volatile Constituents of the Essential Oil of the Peels of Juglans nigra L, Journal of Essential Oil Research 4(5) (1992) 539-541.

[52] G. Buchbauer, L. Jirovetz, M. Wasicky, A. Nikiforov, Headspace Constituents of Fresh Juglans nigra L. Peels, Journal of Essential Oil Research 5 (1993) 455-457.

[53] A. Cakir, S. Kordali, H. Zengin, S. Izumi, T. Hirata, Composition and antifungal activity of essential oils isolated from Hypericum hyssopifolium and Hypericum heterophyllum, Flavour and Fragrance Journal 19(1) (2004) 62-68.

[54] A. Bertoli, L. Pistelli, I. Morelli, G. Spinelli, F. Menichini, Constituents of Hypericum hircinum Oils, Journal of Essential Oil Research 12(5) (2000) 617-620.

[55] A. Bertoli, F. Menichini, M. Mazzetti, G. Spinelli, I. Morelli, Volatile constituents of the leaves and flowers of Hypericum triquetrifolium Turra, Flavour and Fragrance Journal 18(2) (2003) 91-94.

[56] M. Couladis, P. Baziou, P.V. Petrakis, C. Harvala, Essential oil composition of Hypericum perfoliatum L. growing in different locations in Greece, Flavour and Fragrance Journal 16 (2001) 204-206.

[57] N.X. Duñg, P. Van Khiên, H.T. Chiên, P.A. Leclercq, The Essential Oil of Cinnamomum camphora (L.) Sieb. var. linaloolifera from Vietnam, Journal of Essential Oil Research 5(4) (1993) 451-453.

[58] B.M. Mitzner, S. Lemberg, Δ-Terpineol, The Journal of Organic Chemistry 31(6) (1966) 2022-2023.

[59] A. Gören, G. Topçu, G. Bilsel, M. Bilsel, Z. Aydoğmuş, J.M. Pezzuto, The chemical constituents and biological activity of essential oil of Lavandula stoechas ssp. stoechas, Z Naturforsch C J Biosci 57(9-10) (2002) 797-800.

[60] R.T. Jacobs, G.I. Feutrill, J. Meinwald, Defense mechanisms of arthropods. 84. Synthesis of (-)-.alpha.-necrodol and (-)-.beta.-necrodol: novel cyclopentanoid terpenes from a carrion beetle, The Journal of Organic Chemistry 55(13) (1990) 4051-4062.

[61] F. Boukhari, N. Tigrine-Kordjani, B.Y. Meklati, Phytochemical Investigation by Microwave-Assisted Extraction of Essential Oil of the Leaves of Walnut Cultivated in Algeria, Helvetica Chimica Acta 96 (2013) 1168-1175.

[62] P.M. Santos, A.C. Figueiredo, M.M. Oliveira, J.G. Barroso, L.G. Pedro, S.G. Deans, A.K.M. Younus, J.J.C. Scheffer, Essential oils from hairy root cultures and from fruits and roots of Pimpinella anisum, Phytochemistry 48 (1998) 455-460.

[63] F. Muller-Riebau, B.M. Berger, O. Yegen, Chemical composition and fungitoxic properties to phytopathogenic fungi of essential oils of selected aromatic plants growing wild in Turkey, Journal of Agricultural and Food Chemistry 43 (1995) 2262-2266.

[64] S. Sosa, G. Altinier, M. Politi, A. Braca, I. Morelli, R. Della Loggia, Extracts and constituents of Lavandula multifida with topical anti-inflammatory activity, Phytomedicine 12(4) (2005) 271-7.

[65] J. de Pascual-T, E. Caballero, C. Caballero, G. Machin, Constituents of the essential oil of Lavandula latifolia, Phytochemistry 22(4) (1983) 1033-1034.

[66] M. Skoula, C. Abidi, E. Kokkalou, Essential oil variation of Lavandula stoechas L. ssp. stoechas growing wild in crete (Greece), Biochemical Systematics and Ecology 24 (1996) 255-260.

[67] M.C. M.I. García-Vallejo, V. García, J. Sanz, M. Bernabe, A. Velasco-Negueruela, Necrodane (1,2,2,3,4-pentamethylcyclopentane) derivatives in Lavandula luisieri, new compounds to the plant kingdom, Phytochemistry 36(1) (1994) 43-45.

[68] D.P. Costa, E.G. Alves Filho, L. Silva, S.C. Santos, X.S. Passos, M.d.R.R. Silva, J.C. Seraphin, P.H. Ferri, Influence of fruit biotypes on the chemical composition and antifungal activity of the essential oils of Eugenia uniflora leaves, Journal of the Brazilian Chemical Society 21 (2010) 851-858.

[69] D.e. Karasawa, S. Shatar, A. Erdenechimeg, Y. Okamoto, H. Tateba, S. Shimizu, A Study on Mongolian Mints. A New Chemotype from Mentha asiatica Borriss and Constituents of M. arvensis L. and M. piperita L, Journal of Essential Oil Research 7(3) (1995) 255-260.

[70] F. Mueller-Riebau, B. Berger, O. Yegen, Chemical Composition and Fungitoxic Properties to Phytopathogenic Fungi of Essential Oils of Selected Aromatic Plants Growing Wild in Turkey, Journal of Agricultural and Food Chemistry 43(8) (1995) 2262-2266.
