# Supplementary material for: Development of a novel, entirely herbal-based mouthwash effective against common oral bacteria and SARS-CoV-2
Source: BMC Complement Med Ther. 2023 May 1;23:138. doi: 10.1186/s12906-023-03956-3 (PMC10150350; doi:10.1186/s12906-023-03956-3)
Supplement: Supplementary file 5 — Additional file 5. Ingredients of different mouthwashes, categorized by the EWG numbers. Ingredients of different mouthwashes, categorized by the EWG numbers. *This ingredient’s score is higher if used in products intended for use around mouth and on lips due to increased risk of ingestion and absorption. †This ingredient’s score is higher if used in inhalable products (e.g., sprays, powders) because of respiratory concerns. ‡This ingredient’s score is higher if used in products intended for use around mouth, on lips, around the eyes and for damaged skin due to increased risk of ingestion and absorption [23]. [file 12906_2023_3956_MOESM5_ESM.docx]

| **Mouthwash** | **Manufacturer** | **Ingredients** | **EWG number** |
| --- | --- | --- | --- |
| **Sensodyne Cool Mint** | GlaxoSmithKline | Aqua | 1 |
|  |  | Glycerin | 2 |
|  |  | Sorbitol | 1 |
|  |  | Potassium Nitrate | 1 |
|  |  | PEG-60 Hydrogenated Castor Oil | 3 |
|  |  | Poloxamer 407 | 2 |
|  |  | Sodium Benzoate | 3 |
|  |  | Aroma | no data |
|  |  | Disodium Phosphate | 1 |
|  |  | Methylparaben | 4 |
|  |  | Propylparaben | 9 |
|  |  | Sodium Phosphate | 1 |
|  |  | Sodium Fluoride | 4 |
|  |  | Sodium Saccharin | 1 |
|  |  | CI 42090 | 7 |
| **Elmex Sensitive** | Colgate-Palmolive | Aqua | 1 |
|  |  | Propylene Glycol | 3 |
|  |  | PEG-40 Hydrogenated Castor Oil | 3 |
|  |  | Olaflur | no data |
|  |  | Aroma | no data |
|  |  | PVP/Dimethylamino-ethylmethacrylate Polycarbamyl Polyglycol Ester | 1 |
|  |  | Saccharin | 1 |
|  |  | Hydroxyethylcellulose | 1 |
|  |  | Potassium Fluoride | no data |
|  |  | Potassium Hydroxide | 5 |
|  |  | Polyaminopropyl Biguanide | 2 |
| **Corsodyl** | GlaxoSmithKline | Chlorhexidine Digluconate | 4 |
|  |  | Glycerol | 2 |
|  |  | Macrogolglycerol Hydroxystearate | no data |
|  |  | Sorbitol Liquid | 1 |
|  |  | Aniseed Flavour | no data |
|  |  | Purified Water | 1 |
| **Listerine Freshburst** | Johnson & Johnson | Eucalyptol | 1 |
|  |  | Menthol | 1 |
|  |  | Thymol | 1-4* |
|  |  | Alcohol | 1 |
|  |  | Aroma (flavor) | no data |
|  |  | Benzoic Acid | 3 |
|  |  | Green 3 | 5 |
|  |  | Methyl Salicylate | 3 |
|  |  | Poloxamer 407 | 2 |
|  |  | Sodium Benzoate | 3 |
|  |  | Sodium Saccharin | 1 |
|  |  | Sorbitol | 1 |
|  |  | Water | 1 |
|  |  | Yellow 10 | 1 |
| **Colgate Plax Cool Mint** | Colgate-Palmolive | Aqua | 1 |
|  |  | Glycerin | 2 |
|  |  | Propylene Glycol | 3 |
|  |  | Sorbitol | 1 |
|  |  | Poloxamer 407 | 2 |
|  |  | Aroma | no data |
|  |  | Cetylpyridinium Chloride | 5 |
|  |  | Potassium Sorbate | 2 |
|  |  | Sodium Fluoride | 4 |
|  |  | Sodium Saccharin | 1 |
|  |  | Menthol | 1 |
|  |  | CI 42051 | 3 |
| **Oral-B Pro-Expert** | Procter&Gamble | Aqua | 1 |
|  |  | Glycerin | 2 |
|  |  | Aroma | no data |
|  |  | Cetylpyridinium Chloride | 5 |
|  |  | Poloxamer 407 | 2 |
|  |  | Methylparaben | 4 |
|  |  | Sodium Saccharin | 1 |
|  |  | Cinnamal | 5 |
|  |  | Propylparaben | 9 |
|  |  | Eugenol | 3-5† |
|  |  | CI 42090 | 7 |
| **Aquafresh Fresh Mint** | GlaxoSmithKline | Aqua | 1 |
|  |  | Glycerin | 2 |
|  |  | PEG-60 Hydrogenated Castor Oil | 3 |
|  |  | Sodium Citrate | 1 |
|  |  | Aroma | no data |
|  |  | Zinc Chloride | 4 |
|  |  | Cetylpyridinium Chloride | 5 |
|  |  | Sodium Saccharin | 1 |
|  |  | Sodium Fluoride | 4 |
|  |  | Pentasodium Triphosphate | 1 |
| **Cserszömörcés** | Herbária | Cotinus Coggygria Leaf Extract | no data |
|  |  | Thymus Culgaris Flower/Leaf/Stem Extract | 1 |
|  |  | Alcohol | 1 |
|  |  | PEG-40 Hydrogenated Castor Oil | 3 |
|  |  | Aroma | no data |
|  |  | Aqua | 1 |
|  |  | Limonene | 5 |
| **Dontodent** | dm - Drogerie Markt GMBH | Aqua | 1 |
|  |  | Glycerin | 2 |
|  |  | Propylene Glycol | 3 |
|  |  | Xylitol Sodium Fluoride | no data |
|  |  | Cetylpyrinidium Chloride | 5 |
|  |  | Sodium Saccharin | 1 |
|  |  | Mentha Piperita Leaf Extract | 4 |
|  |  | Chamomilla Recutita Flower Extract | 2 |
|  |  | Salvia Officinalis Leaf Extract | 1 |
|  |  | Commiphora Myrrha Resin Extract | 1 |
|  |  | Cocamidopropyl Betaine | 5 |
|  |  | Sodium Benzoate | 3 |
|  |  | Disodium Phosphate | 1 |
|  |  | Lactic Acid | 4 |
|  |  | Citric Acid | 2 |
|  |  | Aroma | no data |
|  |  | Limonene | 5 |
|  |  | Sodium Chloride | 1 |
|  |  | Sodium Sulfate | 1 |
|  |  | CI 13015 | 1 |
|  |  | CI 15510 | 2-5‡ |
|  |  | CI 42051 | 3 |

Additional File 3. Ingredients of different mouthwashes, categorized by the EWG numbers. *This ingredient’s score is higher if used in products intended for use around mouth and on lips due to increased risk of ingestion and absorption. †This ingredient’s score is higher if used in products that are inhalable (e.g., sprays, powders) because of respiratory concerns. ‡This ingredient’s score is higher if used in products intended for use around mouth, on lips, around the eyes and for damaged skin due to increased risk of ingestion and absorption. [23]
