# Supplementary material for: Development of a novel, entirely herbal-based mouthwash effective against common oral bacteria and SARS-CoV-2
Source: BMC Complement Med Ther. 2023 May 1;23:138. doi: 10.1186/s12906-023-03956-3 (PMC10150350; doi:10.1186/s12906-023-03956-3)
Supplement: Supplementary file 7 — Additional file 7. Original image of the silver stained SDS polyacrylamide gel for Fig. 2. [file 12906_2023_3956_MOESM7_ESM.docx]

**a b c d e f g h M**


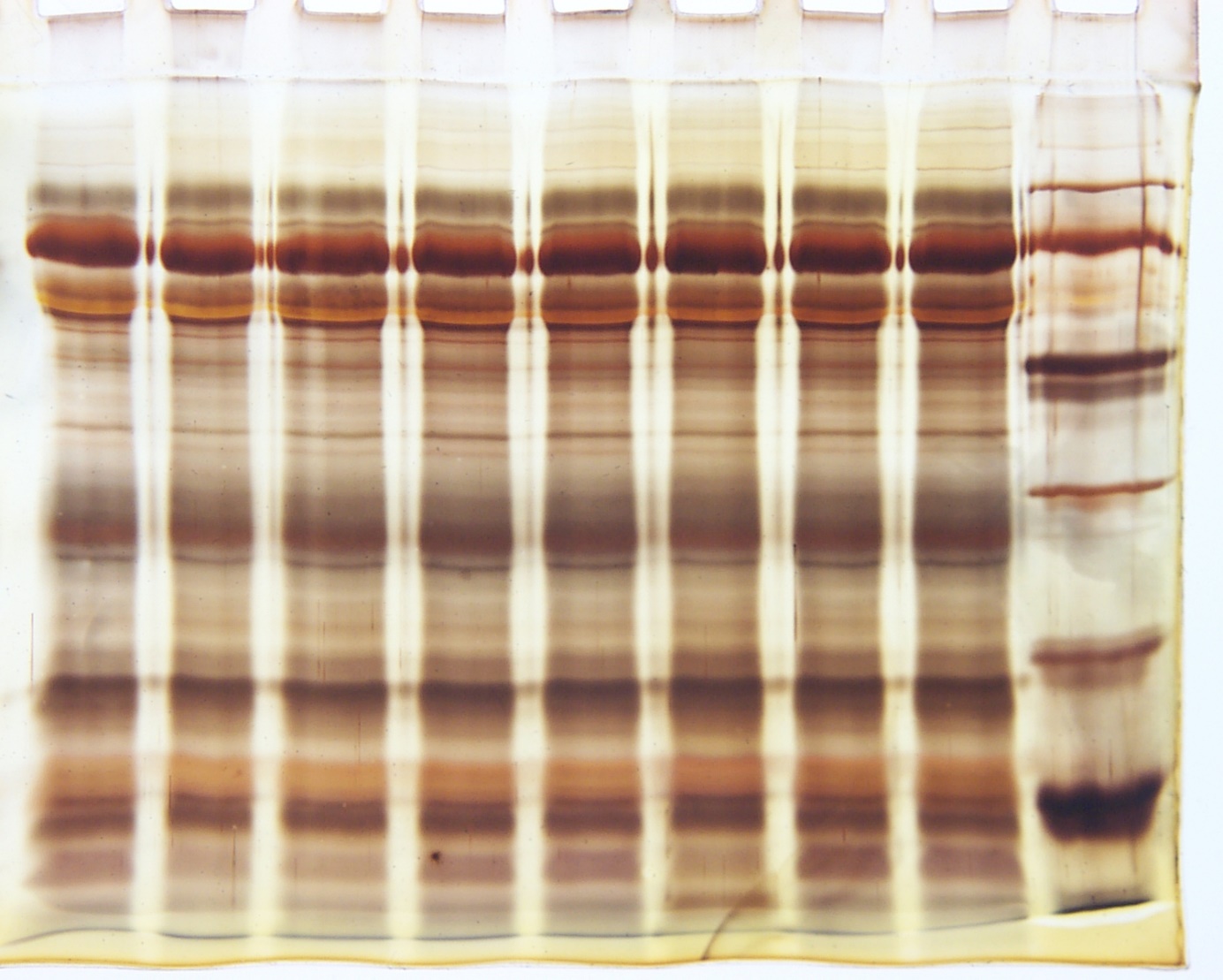


Original image of the silver stained SDS polyacrylamide gel for Figure 2. A. Bold letters on the top indicate the following substances mixed with the saliva samples: a. Herba Dei 4-times diluted, b. Herba Dei 5-times diluted, c. Herba Dei 6.67-times diluted, d. Herba Dei 10-times diluted, e. Herba Dei 20-times diluted, f. PBS, g. 10% ethanol, h. native saliva, M: low molecular weight protein marker. In Figure 2. A, the grey section on the right is a duplicate of the rightmost 3 lanes of the same gel for illustrational purposes.
